# Identification of Novel Reference Genes Using Multiplatform Expression Data and Their Validation for Quantitative Gene Expression Analysis

**DOI:** 10.1371/journal.pone.0006162

**Published:** 2009-07-07

**Authors:** Mi Jeong Kwon, Ensel Oh, Seungmook Lee, Mi Ra Roh, Si Eun Kim, Yangsoon Lee, Yoon-La Choi, Yong-Ho In, Taesung Park, Sang Seok Koh, Young Kee Shin

**Affiliations:** 1 Laboratory of Molecular Pathology, Department of Pharmacy, College of Pharmacy, Seoul National University, Seoul, Korea; 2 Interdiciplinary Program of Bioinformatics, College of Natural Science, Seoul National University, Seoul, Korea; 3 Department of Statistics, College of Natural Science, Seoul National University, Seoul, Korea; 4 BIT center, CT&D Ltd., Seoul, Korea; 5 LG Life Sciences, Ltd., R&D Research Park, Daejeon, Korea; 6 Department of Pathology, Samsung Medical Center, Sungkyunkwan University School of Medicine, Seoul, Korea; 7 Protein Therapeutics Research Center, Korea Research Institute of Bioscience and Biotechnology, Daejeon, Korea; Deutsches Krebsforschungszentrum, Germany

## Abstract

Normalization of mRNA levels using endogenous reference genes (ERGs) is critical for an accurate comparison of gene expression between different samples. Despite the popularity of traditional ERGs (tERGs) such as *GAPDH* and *ACTB*, their expression variability in different tissues or disease status has been reported. Here, we first selected candidate housekeeping genes (HKGs) using human gene expression data from different platforms including EST, SAGE, and microarray, and 13 novel ERGs (nERGs) (*ARL8B, CTBP1, CUL1, DIMT1L, FBXW2, GPBP1, LUC7L2, OAZ1, PAPOLA, SPG21, TRIM27, UBQLN1, ZNF207*) were further identified from these HKGs. The mean coefficient variation (CV) values of nERGs were significantly lower than those of tERGs and the expression level of most nERGs was relatively lower than high expressing tERGs in all dataset. The higher expression stability and lower expression levels of most nERGs were validated in 108 human samples including formalin-fixed paraffin-embedded (FFPE) tissues, frozen tissues and cell lines, through quantitative real-time RT-PCR (qRT-PCR). Furthermore, the optimal number of nERGs required for accurate normalization was as few as two, while four genes were required when using tERGs in FFPE tissues. Most nERGs identified in this study should be better reference genes than tERGs, based on their higher expression stability and fewer numbers needed for normalization when multiple ERGs are required.

## Introduction

Gene expression analysis is becoming more important in diagnostic fields as it allows for the identification of novel biomarkers relevant to diseases. Endogenous reference genes (ERGs) are widely used to normalize the mRNA level in the relative quantification to provide an accurate comparison of gene expression between different samples [1]. Traditional ERGs (tERGs), such as *GAPDH* and *ACTB*, have been used in expression studies without proper validation because of the assumption that they are expressed at constant levels across different samples and regardless of experimental treatments [2], [3]. However, several reports have shown that the expression of tERGs can vary in different tissues and be regulated by experimental treatments or pathological state [2], [4]–[10]. As the use of inappropriate ERGs in relative quantification of gene expression can result in biased expression profiles [4], [11], [12], the selection of proper ERGs is essential for accurate measurement especially in quantitative methods including qRT-PCR, which is a highly sensitive and accurate method [13].

Although there have been a number of previous studies aimed at finding suitable ERGs, most of them have focused on selecting the most stable genes from commonly used ERGs [14]–[17]. Moreover, the identification of novel ERGs (nERGs) has been based primarily on microarray data [10], [18]–[21]. Although short-oligo microarrays such as Affymetrix GeneChips, have the advantage of being highly sensitive in detecting low abundance transcripts (nearly 3–20 transcripts per million (tpm)) [22], they have some disadvantages such as inaccurate cross hybridization between probes and transcripts, differences in hybridization efficiencies between probe sets, limited linear range of signal, and incorrect annotation of transcripts [23], [24]. Therefore, an approach using only microarray data may not be sufficient to identify the most suitable ERGs. Although an ideal universal ERG may not exist [1], [15], a combination of large expression data from different platforms is expected to complement the limitation of each platform [25] and allow for the identification of more suitable ERGs.

Here, we describe an algorithm for the identification of nERGs using the publicly available human gene expression datasets in addition to in-house microarray data. The expression of selected nERGs in datasets was validated by qRT-PCR in 108 human samples and their expression stability was compared to that of tERGs.

## Materials and Methods

### Ethics Statement

This study was approved by the institutional review board of Samsung Medical Center. Patients' written informed consent was not required to be obtained as the institutional review board approved the use of human tissues in this study without patients' consent with the reason that the data were analyzed anonymously and patients could not be identified.

### EST, SAGE, microarray gene expression dataset construction

**Figure 1:**
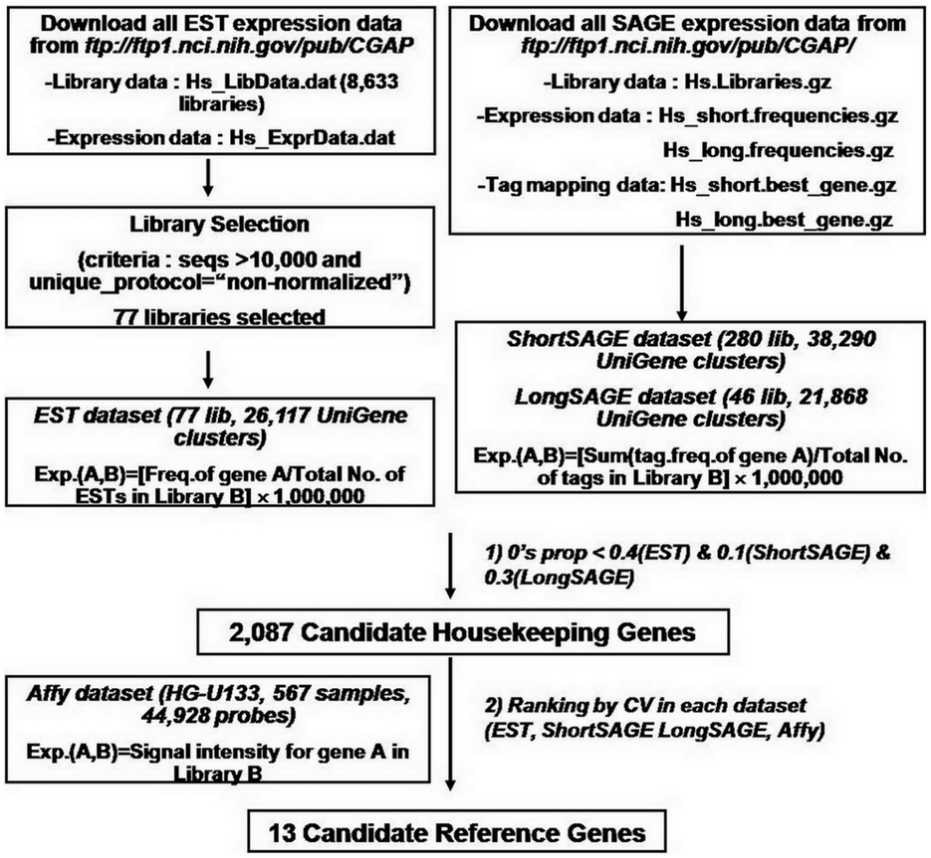
Flowchart of the methodology for identification of nERGs. 2,087 candidate HKGs were first identified by selecting the genes meeting the following criteria: 0's prop <0.4 in EST, <0.1 in shortSAGE and <0.3 in longSAGE. 0's prop represents 0's proportion (number of tissues in which the gene is not expressed/total number of tissues, 0≤0's prop≤1). Among the candidate 2,087 HKGs, 13 nERGs with the lowest CVs were further identified by selecting the genes common to all four datasets among the genes with the 400 lowest CVs (approximately 20% of candidate HKGs).

EST and SAGE human gene expression data were collected from the publicly available Cancer Genome Anatomy Project (CGAP) site (http://cgap.nci.nih.gov/). Microarray data was obtained from the Cancer Genome Expression Database of LG Life Sciences, which is based on the Affymetrix HG-U133 (for the samples included in microarray data, see Table S1)[26]. A detailed description of each dataset construction is provided in the Supplementary Materials and Methods (Text S1) and shown schematically in Figure 1.

### Algorithm for the identification of candidate HKGs and 13 nERGs

The methodology used to identify nERGs is outlined in Figure 1. First, to identify nERGs, we searched for candidate HKGs whose expression is detected in most tissues using 0's proportion in EST and SAGE datasets. 0's proportion is defined as the proportion of different tissues in which a given gene is not expressed and was calculated as follows:
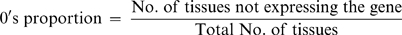



In this equation a value of 0 indicates that the gene is expressed in all tissues, and a value of 1 indicates that the gene is not expressed in any tissue. If a gene was expressed in any one library of multiple libraries corresponding to the same tissue, that gene was considered to be expressed in that tissue. A total of 2,087 candidate HKGs with low 0's proportions in all three datasets were selected (Table S2). Cut-off values for 0's proportion in each dataset were determined to include the majority of the 575 HKGs identified from a previous Affymetrix U95A chip [27] (see Figure 1, Text S1). Affymetrix (Affy) data for 5,317 probe sets corresponding to 2,087 HKGs were obtained. Mean gene expression and CV (%) for each gene were calculated in each dataset. The Student's t-test was used to assess whether the mean gene expression between candidate HKGs and non-HKGs was statistically different. When this set of 2,087 HKGs was classified functionally using the Functional Classification Catalogue (FunCat, Version 2.0) [28], known functions were available for only 1,318 genes of them (for details, see Text S1). For CpG island analysis, sequences upstream of the annotated transcription start site of RefSeq genes were obtained from the UCSC site and CpG islands in their upstream sequences were searched using the CpGIE software [29]. The statistical significance of differences in the frequency of genes with CpG islands between HKGs and non-HKGs was determined using Z-test (R program, http://www.R-project.org). *P*<0.05 was considered to be significantly different.

Among the 2,087 HKGs, potential ERGs were further identified according to the following process: 1) the CV was calculated for each UniGene cluster and 2) the genes in each dataset were ranked by ascending CV values. Among the first 400 genes with the lowest CVs (approximately 20% of 2,087 HKGs), we found 13 nERGs common to all four datasets (i.e. EST, LongSAGE, ShortSAGE, and Affy). The CV values between tERGs and nERGs were compared using Wilcoxon rank sum test.

### Genomic variations of nERGs and tERGs

The Database of Genomic Variants (http://projects.tcag.ca/variation/) was used to search for genomic variations of the 2,087 HKGs, including nERGs and tERGs.

### RNA preparation from human frozen, FFPE tissues and human cancer cell lines and qRT-PCR

The Department of Pathology at Samsung Medical Center provided 26 human frozen tissues and 60 FFPE tissues and the Korea Cell Line Bank (KCLB) or ATCC provided 22 human cancer cell lines (Table S3). The 60 FFPE tissues consisted of 10 breast cancers, 8 normal stomach, 9 stomach cancers, 10 normal ovaries, 4 ovarian adenomas, 9 ovarian borderline tumors and 10 ovarian cancers.

Total RNA isolation and cDNA synthesis from human samples are described in detail in the Supplementary Materials and Methods (Text S1). Total RNA from frozen tissues and cell lines was isolated using Trizol (Invitrogen Life Technologies). The inclusion criteria for the RNA samples were A260/280≥1.80 and rRNA (28S/18S) ratio≥1.0. The integrity of RNA from frozen tissues and cell lines was assessed using an Agilent Bioanalyzer 2100 (Agilent Technologies, Palo Alto, CA). Paradise Whole Transcript RT Reagent system (Arcturus, CA, USA) was used for RNA isolation and RT of FFPE tissues.

PCR primers and Universal Probe Library (UPL) numbers for this study are provided in Table 1. Primers and probes for each gene were designed to have a short amplicon size. All PCR reactions were performed in a Lightcycler 2.0 (Roche Applied Science) according to standard procedures (for details, see Text S1). PCR efficiency for each gene in frozen tissues and cell lines was determined by both the serial dilution method using Lightcycler 4.0 software and estimation using the LinRegPCR program [30] (Table 1, Text S1). To minimize experimental variation, the same gene in different samples was tested in the same PCR run.

**Table 1:** Real-time PCR primers and Taqman probes used in this study.

Gene Symbol	Title	Accession number	Probe* (UPL Probe No.)	Primer	Amplicon size (bp)	PCR efficiency (dilution)**	PCR efficiency (LinRegPCR)***
GAPDH	Glyceraldehyde-3-phosphate dehydrogenase	NM_002046	60	Left 18 agccacatcgctcagaca	66	1.899	1.735±0.048 (137)
				Right 19 gcccaatacgaccaaatcc			
ACTB	Actin, beta	NM_001101	64	Left 18 ccaaccgcgagaagatga	97	2.038	1.491±0.034 (137)
				Right 20 ccagaggcgtacagggatag			
B2M	Beta-2-microglobulin	NM_004048	42	Left 19 ttctggcctggaggctatc	86	1.868	1.717±0.068(140)
				Right 23 tcaggaaatttgactttccattc			
PPIA	Peptidylprolyl isomerase A (cyclophilin A)	NM_021130	Specific Probe#	Left 22 catctgcactgccaagactgag	326	1.877	1.773±0.058(142)
				Right 19 tgcaatccagctaggcatg			
HPRT1	Hypoxanthine phosphoribosyltransferase 1	NM_000194	73	Left 24 tgaccttgatttattttgcatacc	102	1.800	1.771±0.024 (143)
				Right 20 cgagcaagacgttcagtcct			
HMBS	Hydroxymethylbilane synthase	NM_000190	26	Left 18 tgtggtgggaaccagctc	92	1.954	1.431±0.031(143)
				Right 19 tgttgaggtttccccgaat			
TBP	TATA box binding protein	NM_003194	3	Left 20 gctggcccatagtgatcttt	60	1.826	1.447±0.038(142)
				Right 21 cttcacacgccaagaaacagt			
H6PD	Hexose-6-phosphate dehydrogenase	NM_004285	89	Left 23 tggagatcatcatgaaagagacc	74	1.874	1.832±0.026 (64)
				Right 20 gcgaatgacaccgtactcct			
ZNF207	Zinc finger protein 207	NM_003457	27	Left 21 ctgtttcctagcacagcacaa	65	1.869	1.648±0.018 (142)
				Right 23 ggtttgaaatctgtaccaacagg			
OAZ1	Ornithine decarboxylase antizyme 1	NM_004152	74	Left 18 caccatgccgctcctaag	67	2.068	1.498±0.059(142)
				Right 20 gagggagaccctggaactct			
LUC7L2	LUC7-like 2 (S. cerevisiae)	NM_016019	85	Left 20 cgatcacacagcaagaatcc	60	1.829	1.709±0.047 (143)
				Right 19 agatcgatgtctgcgatgc			
CTBP1	C-terminal binding protein 1	NM_001012614 NM_001328	77	Left 18 actgcgtgaccctgcact	86	2.064	1.651±0.055 (141)
				Right 18 gccccttgtctcatctgc			
TRIM27	Tripartite motif containing 27	NM_006510 NM_030950	7	Left 19 caggcacgagctgaactct	71	1.908	1.693±0.034 (143)
				Right 20 agctgctcaaactcccaaac			
GPBP1	GC-rich promoter binding protein 1	NM_022913	4	Left 21 tcacttgaggcagaacacaga	75	1.844	1.715±0.031 (141)
				Right 23 agcacatgtttcatcattttcac			
UBQLN1	Ubiquilin 1	NM_013438	73	Left 20 gaatcctgaccttgctgcac	92	1.864	1.723±0.027(143)
				Right 21 ttgggagctgttgtctcattt			
ARL8B	ADP-ribosylation factor-like 8B	NM_018184	82	Left 19 aagcatgtgggagcggtat	66	1.838	1.499±0.074(139)
				Right 22 cgatctgcagcatctatcatgt			
PAPOLA	Poly(A) polymerase alpha	NM_032632	78	Left 21 gctacgaagaccagtccattg	91	1.830	1.509±0.032(141)
				Right 20 tgttggtcacagatgctgct			
CUL1	Cullin 1	NM_003592	65	Left 18 gcgaggtcctcactcagc	86	1.810	1.695±0.027(139)
				Right 26 ttctttctcaattagaatgtcaatgc			
DIMT1L	DIM1 dimethyladenosine transferase 1-like (S. cerevisiae)	NM_014473	77	Left 27 tccagtgttgtaaggatagaacctaag	75	1.906	1.655±0.037(141)
				Right 23 ccttactagaccatcccattcct			
FBXW2	F-box and WD-40 domain protein 2	NM_012164	3	Left 19 cggctctgcagacttcact	111	1.891	1.638±0.02(142)
				Right 22 ttgcacttctgcaaaactacct			
SPG21	Spastic paraplegia 21 (autosomal recessive, Mast syndrome)	NM_016630	21	Left 20 gatgtctttttccggcagat	88	1.826	1.636±0.021(142)
				Right 21 cgagatggtcccaataaactg			

* UPL probes were designed using Probe Finder in the Universal Probe Library (UPL) Assay Design Center (Roche Applied Science, Mannheim, Germany).

** PCR efficiency for each gene was determined by using serial dilutions of cDNA from MKN 74 cells for PCR and then calculating the efficiency using the Roche Lightcycler software 4.0.

*** PCR efficiencies of 48 samples in triplicate were calculated using LinRegPCR (Ramakers et al. Neurosci Lett, 2003. **339**(1): p. 62–6.).

# **F-**ttcttgctggtcttgccat**T**cctgga-**p** (T, TAMRA-labeled; F, FAM-labeled; P, Phosphate).

### Gene expression stability analysis in qRT-PCR

To analyze gene expression stability, we used the geNorm v.3.4 [1] and NormFinder software [31]. Cp values were converted into relative quantities for analysis with geNorm and NormFinder, taking into consideration the PCR efficiencies of the genes, as shown in Table 1 (for details, see Text S1). The optimal number of ERGs for normalization was also determined using the geNorm program. The statistical differences in gene expression stability values between nERGs and tERGs were determined using Wilcoxon rank sum test. The Pearson and Spearman correlation between the gene expression stability calculated by geNorm or NormFinder (M for geNorm and S for NormFinder) and the CV calculated in each dataset were analyzed using R statistical software. *P*<0.05 was considered to be significant.

## Results

### Identification and characterization of 2,087 candidate HKGs

**Figure 2:**
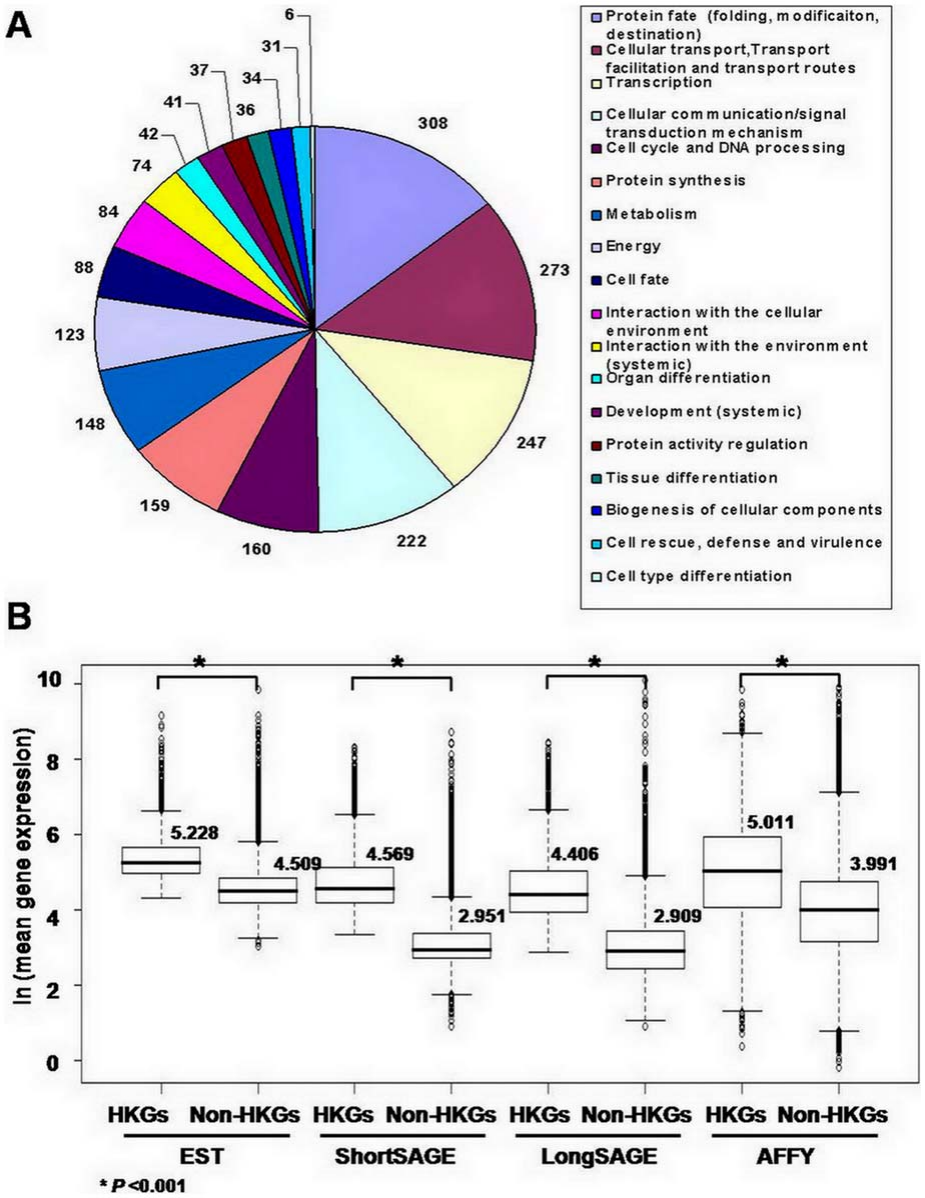
Characterization of candidate HKGs identified in this study. (A) The functional distribution of candidate HKGs was classified by FunCat. Among the 2,087 UniGene clusters, a total of 1,605 UniGene clusters which have GO terms under biological processes were classified according to their major functional categories using FunCat (version 2.0) by mapping the GO terms to FunCat categories. A total of 1,318 UniGene clusters were classified by FunCat. The number of UniGene clusters belonging to each category is presented in A. In some cases, UniGene clusters mapped to two or more FunCat categories. (B) Comparison of gene expression between candidate HKGs and non-HKGs in each dataset. Box and Whisker plots provide a simple description of a distribution of values by depicting the 25^th^ and 75^th^ percentile values as the bottom and top of a box, respectively. The Y axis represents the natural logarithm transformed mean gene expression levels. The median expression values of HKGs and non-HKGs are marked by horizontal lines in the boxes and the values are provided to the right of each box. *P<0.001.

2,087 candidate HKGs were first identified using 0's proportion in each dataset (Figure 1, Table S2). According to the functional classification by FunCat [28], genes with a variety of basic cellular functions were included in this list. In particular, proteins mediating protein fate (23%) and cellular transport (21%) had the highest frequency (Figure 2A). This is in contrast to the previously reported classifications of HKGs, in which metabolic and ribosomal proteins were enriched [27], [32]. We compared the frequency of genes with CpG islands in the upstream sequences of transcription start sites in HKGs relative to non-HKGs. Most HKGs (70%) were found to possess a CpG island within 1,000 bp from the transcription start site, consistent with previous studies [33], [34], while fewer CpG islands were found in the upstream sequences of non-HKGs (*P*<0.001) (Table 2). Mean expression level of HKGs was significantly higher than that of non-HKGs in all datasets (*P*<0.001) (Figure 2B), also consistent with previous work [27] (for detailed description on the expression of 2,087 HKGs in 4 datasets, see Text S2). CV values of the 2,087 genes showed a poor correlation between the four datasets, whereas gene expression showed a relatively higher correlation (Table S4).

**Table 2:** Comparison of the proportion of genes with CpG islands in sequences upstream of the transcription start site in HKGs and non-HKGs.

	HKGs (n = 2,002)*		Non-HKGs (n = 14,848)**		
Upstream sequences (bp)	No. of genes	Frequency of genes with CpG islands (%)	No. of genes	Frequency of genes with CpG islands (%)	*P* value
5,000	1,516	76	8,036	54	<0.001
2,000	1,456	73	7,410	50	<0.001
1,000	1,410	70	7,048	47	<0.001

* Of 2,087 genes, 2,002 UniGene clusters corresponded to upstream sequences downloaded from the UCSC site (http://hgdownload.cse.ucsc.edu/goldenPath/hg18/bigZips/).

** A total of 16,850 UniGene clusters corresponded to upstream sequences downloaded from the UCSC site and the number of non-HKGs was calculated by subtracting the 2,002 HKGs from the total 16,850.

CpG island criteria: length≥500 bp, % GC≥55, CpG o/e ratio≥0.65.

### Identification and characterization of 13 nERGs

A total of 13 nERGs common to the four datasets (*ZNF207, OAZ1, LUC7L2, CTBP1, TRIM27, GPBP1, ARL8B, UBQLN1, PAPOLA, CUL1, DIMT1L, FBXW2*, and *SPG21*, Table 3) were identified from 2,087 HKGs. The highest proportion (5/13) (*ZNF207, OAZ1, CTBP1, PAPOLA, FBXW2*) of genes were genes involved in cellular metabolism. CpG islands were found in the upstream region from transcription start site of all 13 nERGs (Table S5).

**Table 3:** nERGs identified from four datasets.

UniGene cluster	Gene Symbol	Gene Title	EST			SHORT SAGE			LONG SAGE			Affymetrix		Gene Ontology	
			Mean	CV	0's P	Mean	CV	0's P	Mean	CV	0's P	Mean	CV	Biological Process	Molecular Function
Hs.446427	OAZ1	Ornithine decarboxylase antizyme 1	673.68	62.71	0.069	576.39	55.94	0	444.92	41.01	0	1860.9	22.07	Polyamine biosynthesis	Ornithine decarboxylase inhibitor activity
Hs.9589	UBQLN1	Ubiquilin 1	111.34	60.19	0.31	75.93	61.3	0.036	71.77	49.75	0.111	919.32	26.81		Kinase binding
Hs.444279	GPBP1	GC-rich promoter binding protein 1	132.98	56.92	0.241	63.11	61.65	0	80.72	51.79	0.111	746.56	26.6		
Hs.208597	CTBP1	C-terminal binding protein 1	136.74	48.53	0.138	213.99	62.01	0	112.96	50.6	0	481.51	24.72	Negative regulation of cell proliferation; Protein phosphorylation; Viral genome replication	Protein C-terminus binding; Transcription factor binding
Hs.253726	PAPOLA	Poly(A) polymerase alpha	216.15	65.36	0.172	118.24	58.02	0	89.5	50.88	0	451.23	27.68	mRNA polyadenylation	RNA binding
Hs.250009	ARL8B	ADP-ribosylation factor-like 8B	132.27	55.79	0.379	134.21	61.55	0	59.19	55.14	0.111	418.28	26.81	Chromosome segregation	α-tubulin binding;β-tubulin binding; GDP binding; GTP binding; GTPase activity
Hs.242458	SPG21	Spastic paraplegia 21 (autosomal recessive, Mast syndrome)	120.35	59.44	0.31	76.41	57.83	0.036	73.3	49.12	0	415.64	29.35	Antigen receptor-mediated signaling pathway	CD4 receptor binding
Hs.530118	LUC7L2	LUC7-like 2 (S. cerevisiae)	132.76	59.55	0.172	74.65	65.41	0	57.21	50.39	0.111	386.79	22.9		
Hs.500775	ZNF207	Zinc finger protein 207	233.29	62.27	0.034	165.68	56.77	0	154.32	52.88	0.111	358.69	18.38	Regulation of transcription, DNA-dependent	Transcription factor activity; Zinc ion binding
Hs.533222	DIMT1L	DIM1 dimethyladenosine transferase 1-like (S. cerevisiae)	129.17	69.14	0.379	42.41	60.55	0.071	36.87	44.13	0.111	164.72	28.3		
Hs.440382	TRIM27	Tripartite motif containing 27	155.33	68.54	0.172	80.66	63.06	0	67.84	45.41	0.111	163.6	26.3	Cell proliferation; Spermatogenesis	Metal ion binding; Transmembrane receptor protein tyrosine kinase activity
Hs.146806	CUL1	Culin 1	120.27	57.5	0.207	69.33	65.76	0.036	76.43	55	0.111	156.47	27.78	Cell cycle arrest; G1/S transition of mitotic cell cycle; Induction of apoptosis by intracellular signals; Negative regulation of cell proliferation	·Protein binding
Hs.494985	FBXW2	F-box and WD-40 domain protein 2	97.45	68.65	0.379	40.27	58.47	0	23.54	51.61	0.111	69.32	28.36	Proteolysis	Protein binding; ubiquitin conjugating enzyme activity; ubiquitin-protein ligase activity

Mean: Mean gene expression, CV: Coefficient of Variation (%), 0's P: 0's proportion, GO terms were searched in the Gene Ontology site (http://www.geneontology.org/).

The gene expression for each of the 13 nERGs showed a significant correlation between datasets (*P*<0.001, Table S6) with high Pearson correlation coefficients (>0.8), although the Spearman correlations of EST versus Affymetrix (0.374, *P* = 0.206) and shortSAGE versus Affymetrix (0.511, *P* = 0.076) were not significant. CVs between the datasets were not significantly correlated (*P*>0.05, Table S6).

To further confirm the suitability of the nERGs, gene copy number variations (CNVs) of the nERGs, which can affect the gene expression, were investigated by searching the Database of Genomic Variants. As shown in Table 4, only *OAZ1* and *DIMT1L* were found to be located in a chromosome region where CNVs were reported, indicating their expression might be deregulated by genomic aberrations.

**Table 4:** Gene copy number variations of nERGs and tERGs.

nERGs					tERGs				
Gene Symbol	Genomic location*	Genomic Variation**			Gene symbol	Genomic location*	Genomic Variation**		
		Variation type	Locus ID	References			Variation type	Locus ID	References
ZNF207	17q11.2				RPLP0	12q24.23			
OAZ1	19p13.3	Copy number	3199	Wong et al (2007)	ACTB	7p22.1	Copy number	1487	Wong et al (2007)
LUC7L2	7q34				PPIA	7p13			
CTBP1	4p16.3				GAPDH	12p13.31	Copy number	2368	Redon et al (2006)
TRIM27	6p22.1				PGK1	Xq21.1	Copy number	3567	Iafrate et al (2004)
GPBP1	5q11.2				B2M	15q21.1	Copy number	2773	Redon et al. (2006)
UBQLN1	9q21.33				GUSB	7q11.21			
ARL8B	3p26.1				HPRT1	Xq26.2			
PAPOLA	14q32.2				TBP	6q27	Copy number	1479	Redon et al (2006)
CUL1	7q36.1				TFRC	3q29	Copy number Copy number	753	Iafrate et al (2004) Redon et al. (2006)
DIMT1L	5q12.1	Copy number	1137	Redon et al (2006)	HMBS	11q23.3			
FBXW2	9q33.2				H6PD	1p36.22			
SPG21	15q22.31				ALAS1	3p21.2	Copy number	590	Wong et al (2007)

* Genomic location was found using Ensembl (http://www.ensembl.org/index.html).

** Genomic variations were found using the Database of Genomic Variants (http://projects.tcag.ca/variation/, Human Genome Assembly Build 36 (hg18)).

### Comparison of tERGs and nERGs in dataset

**Figure 3:**
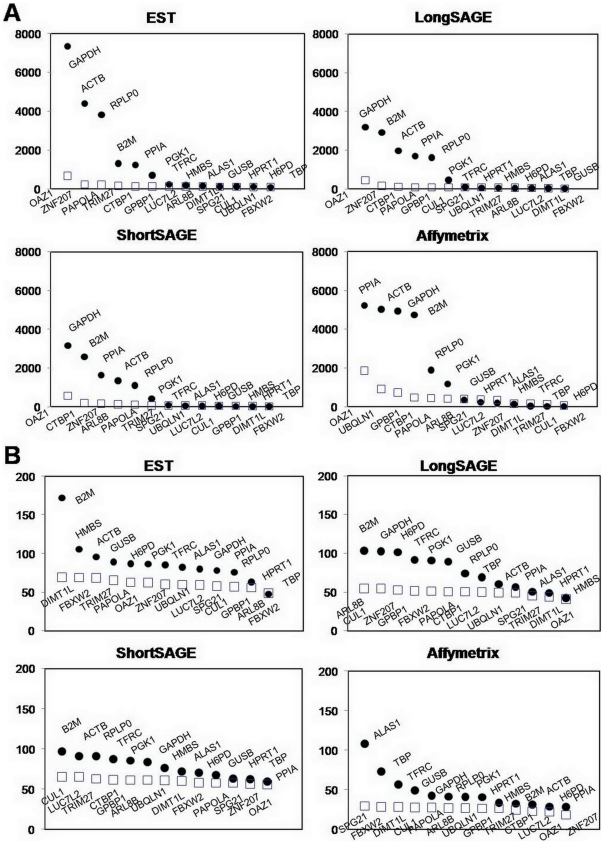
Comparison of gene expression and CV between nERGs and tERGs in each dataset. (A) Comparison of gene expression between 13 nERGs and 13 tERGs. (B) Comparison of CV between 13 nERGs and 13 tERGs. Empty squares represent nERGs identified in this study and circles represent the tERGs.

We compared the 13 nERGs with 13 commonly used tERGs: *GAPDH, ACTB, HPRT1, PPIA, B2M, TBP, HMBS, RPLP0, PGK1, GUSB, TFRC, H6PD*, and *ALAS1*. The mean expression of the nERGs was relatively lower than the highly expressed tERGs, including *GAPDH, ACTB, B2M*, and *PPIA*, in all datasets and was expressed at levels similar to those tERGs, which had lower expression levels (Figure 3, Table S7). With respect to variation, most of the tERGs showed relatively higher variation than the nERGs and the mean CV values of nERGs were significantly lower than those of tERGs (*P*<0.001 in EST, ShortSAGE, and Affymetrix, *P* = 0.003 in LongSAGE, Table S8), supporting the notion that the identified nERGs are generally expressed more stably and at relatively lower levels than tERGs.

A search for genomic variation of tERGs revealed that many of them (*ACTB, GAPDH, PGK1, B2M, TBP, TFRC, ALAS1*) were located in the genomic locus where CNVs are known, whereas only 2 genes among the nERGs were found in those regions (Table 4), suggesting that the higher expression variation of some tERGs might be due in part to CNVs.

### Validation of nERGs by qRT-PCR

To validate both the level and stability of gene expression of nERGs selected from the four datasets, the expression of 13 nERGs and 8 tERGs was measured in a total of 108 human samples, including 26 frozen tissues, 22 cancer cell lines and 60 FFPE tissues, by qRT-PCR using Taqman probes (refer to Text S1 for an explanation for why 8 tERGs among 13 tERGs were chosen for qRT-PCR). Except *PPIA*, a small amplicon for each gene was designed for the reliable measurement of its expression, especially in FFPE tissues where RNA from these samples is frequently degraded. When the PCR efficiency of each gene was determined using the serial dilution method, each gene was amplified at 90–100% efficiency (Table 1). The CVs of Cp values confirmed that the between-assay precision in two or three repeats was within 5% (data not shown).

**Figure 4:**
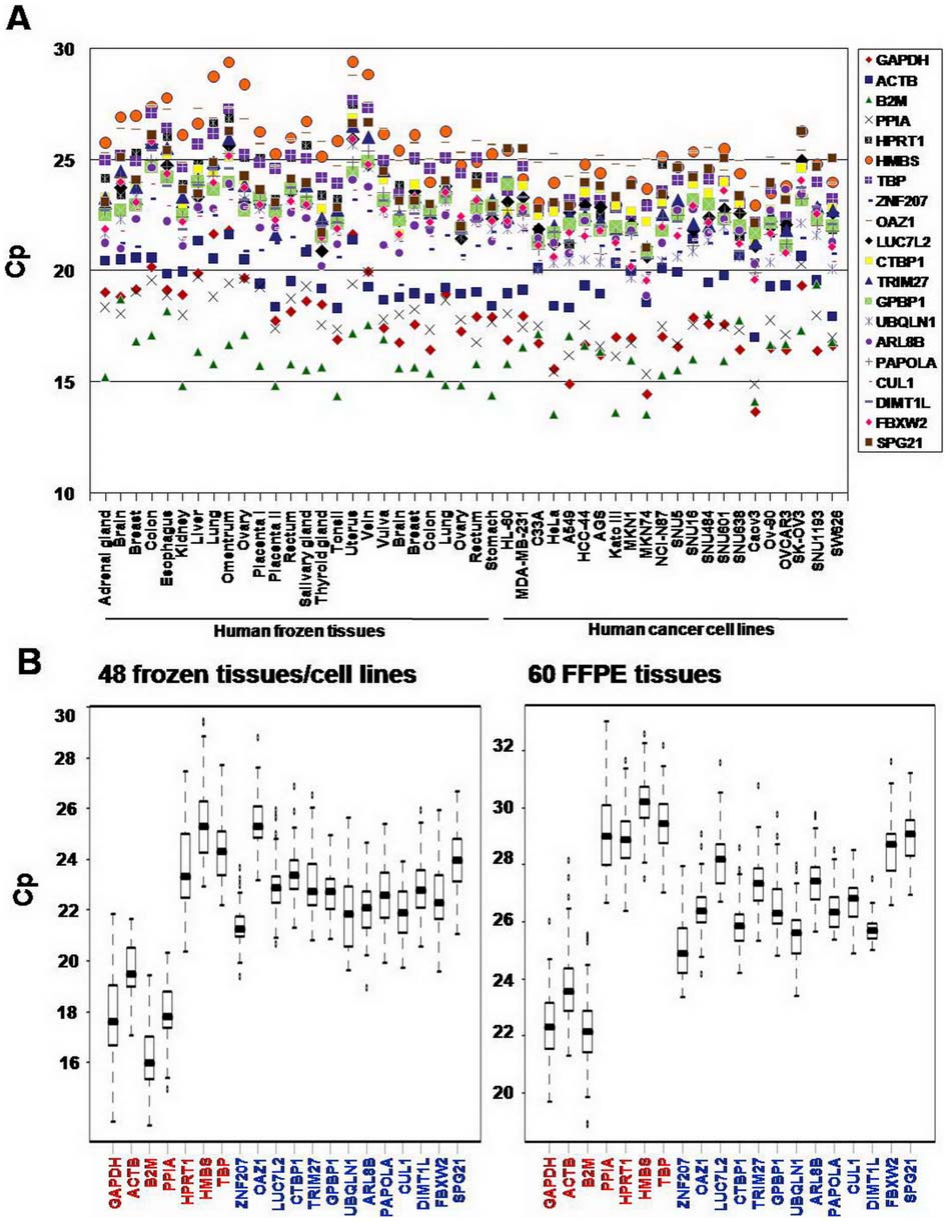
The distribution of expression levels of 13 nERGs and 7 tERGs determined by qRT-PCR using Taqman probes in human samples. (A) The distribution of mRNA levels of tested ERGs in 48 samples, including frozen tissues and cancer cell lines. (B) The mRNA levels of tERGs (red) and nERGs (blue) in Cp values over all 48 samples (left) and 60 FFPE tissues (right). Values are given as “Crossing point” (Cp) values. All measurements of qRT-PCR were repeated three times for frozen tissues and cell lines and twice for FFPE tissues and mean “crossing point” (Cp) values of repeats were calculated. Box and Whisker plots provide a simple description of the distribution of values by depicting the 25^th^ and 75^th^ percentile values as the bottom and top of the box, respectively. The median value is marked by a line within the box and the minimum and maximum values are depicted by error bars, or whiskers, protruding from the box.

First, the expression profiles of these genes in each of the 48 samples, including frozen tissues and cancer cell lines, are presented in Figure 4A. The 13 nERGs were constitutively expressed in all 48 samples. Seven tERGs showed a wide range of expression (Cp: 13.52 to 29.39), but *H6PD* was not widely expressed in frozen tissues and this gene was consequently excluded from subsequent calculations. The Cp values of 13 nERGs ranged from 18.90 to 28.79 (Figure 4B). tERGs could be divided into highly expressed genes (median <20 cycles) and lowly expressed genes (median>23 cycles). Highly expressed genes included *B2M, PPIA, GAPDH, ACTB* and lowly expressed genes consisted of *HPRT1, TBP* and *HMBS*. Of the nERGs, 12 genes displayed an intermediate expression level between the highly expressed and the lowly expressed tERGs (Figure 4B). The mean expression level of nERGs was significantly lower than that of highly expressed tERGs, whereas it was significantly higher than that of lowly expressed tERGs (*P*<0.001, Table S9). *ZNF207* was the most highly expressed gene, followed by *UBQLN1* and *CUL1*. *OAZ1* was the gene with the weakest expression.

We further investigated the expression of the 13 nERGs by qRT-PCR in 60 FFPE tissues to test whether the nERGs could be used in such tissues showing the significant degradation of mRNA. Except *DIMT1L*, the expression of all genes was measurable in all 60 samples. The Cp range for tERGs was 18.85 to 33.02 and for nERGs was 23.33 to 31.58 (Figure 4B). *DIMT1L* was not amplified in 5 samples and therefore was excluded from further expression stability analysis. The expression pattern in the FFPE tissues was similar to that of previous 48 samples (26 frozen tissues and 22 cancer lines) despite the discrepancy in sample types. Remarkably, *PPIA* expression which was detected at high level in frozen tissues/cell lines was observed at markedly decreased level in FFPE tissues. This observation might be due to the long amplicon size of *PPIA* (326 bp), whereas the amplicon size of other genes is small ranging from 60 to 110 bp (Table 1), indicating that small size of amplicon is required for the detection of gene expression in FFPE tissues in which RNA is frequently degraded.

### Gene expression stability of nERGs

We first assessed the gene expression stability (detailed in Text S1) in 48 samples, including 26 frozen tissues and 22 cell lines based on qRT-PCR using two programs, geNorm and NormFinder. All genes tested displayed relatively high expression stability with low M values (<0.9), which were below the default limit of 1.5 in geNorm (Table 5a). *GPBP1* and *CUL1* were identified as the two most stable genes. *B2M* was the least stable gene and had the highest M value (0.888), followed by *ACTB* (0.843), *HMBS* (0.815), and *GAPDH* (0.793). When calculated by NormFinder, *TBP* and *PAPOLA* were the two most stable genes (i.e. having the lowest S values) (Table 5a). Similar to the results from geNorm, tERGs including *B2M, ACTB, GAPDH, HMBS* and *HPRT1*, were found to have less stable expression than nERGs. The mean gene expression stability values in nERGs by geNorm and NormFinder were significantly lower than those in tERGs in both 48 samples and 60 FFPE tissues (*P*<0.05, Tables S10). Both analyses demonstrated that most nERGs showed relatively higher expression stability compared to tERGs, suggesting that nERGs are more suitable for normalization. Moreover, even when gene expression stability was analyzed with relative expression level calculated by PCR efficiencies using the LinRegPCR program, most of nERGs showed a more stable expression than tERGs (data not shown).

**Table 5:** nERGs and tERGs ranked according to their expression stability, as calculated by the two programs, geNorm and NormFinder, based on qRT-PCR data in 48 frozen tissues/cell lines and 60 FFPE tissues.

(a) 48 frozen tissues/cell lines				(b) 60 FFPE tissues			
GeNorm		NormFinder		GeNorm		NormFinder	
Gene Symbol	Average expression stability M	Gene Symbol	Stability value S	Gene Symbol	Average expression stability M	Gene Symbol	Stability value S
GPBP1	0.496	TBP	0.276	GPBP1	0.409	ARL8B	0.233
CUL1		PAPOLA	0.28	PAPOLA		LUC7L2	0.235
PAPOLA	0.536	CUL1	0.287	ARL8B	0.437	OAZ1	0.247
TBP	0.548	LUC7L2	0.29	CTBP1	0.454	CTBP1	0.251
LUC7L2	0.565	CTBP1	0.312	LUC7L2	0.483	UBQLN1	0.273
TRIM27	0.585	GPBP1	0.317	SPG21	0.509	SPG21	0.28
FBXW2	0.597	TRIM27	0.317	FBXW2	0.528	FBXW2	0.286
CTBP1	0.608	FBXW2	0.329	OAZ1	0.545	PAPOLA	0.29
UBQLN1	0.623	DIMT1L	0.364	UBQLN1	0.555	TRIM27	0.327
DIMT1L	0.637	PPIA	0.383	TRIM27	0.567	GPBP1	0.345
PPIA	0.661	UBQLN1	0.398	TBP	0.58	HPRT1	0.368
OAZ1	0.682	OAZ1	0.438	CUL1	0.593	CUL1	0.383
ZNF207	0.709	ARL8B	0.494	HPRT1	0.609	TBP	0.402
ARL8B	0.731	SPG21	0.502	ZNF207	0.625	HMBS	0.407
SPG21	0.749	ZNF207	0.502	HMBS	0.641	ZNF207	0.44
HPRT1	0.77	HPRT1	0.516	GAPDH	0.668	PPIA	0.461
GAPDH	0.793	HMBS	0.587	PPIA	0.692	GAPDH	0.527
HMBS	0.815	GAPDH	0.591	B2M	0.715	B2M	0.53
ACTB	0.843	ACTB	0.618	ACTB	0.737	ACTB	0.541
B2M	0.888	B2M	0.815				

Low average expression stability value M and stability value S indicate the high expression stability.

Pearson correlation analysis revealed the higher concordance of both M and S values with CVs in the EST and shortSAGE (Table 6) than in the Affymetrix. High correlation between M and S (Pearson correlation: 0.953, *P*<0.001) was also observed, indicating that both analyses produced similar results.

**Table 6:** Correlation between gene expression stability of nERGs and tERGs from qRT-PCR data and CV from each dataset.

**48 frozen tissues/cell lines**									
	EST-M	EST-S	ShortSAGE-M	ShortSAGE-S	LongSAGE-M	LongSAGE-S	Affy-M	Affy-S	M-S
Pearson	0.676	0.792	0.659	0.75	0.427	0.561	0.039	0.017	0.953
*P* value	0.001	<0.001	0.002	<0.001	0.061	0.01	0.869	0.944	<0.001
Spearman	0.589	0.605	0.277	0.268	0.092	0.105	0.424	0.357	0.955
*P* value	0.006	0.005	0.237	0.254	0.701	0.661	0.063	0.123	<0.001
**60 FFPE tissues**									
	EST-M	EST-S	ShortSAGE-M	ShortSAGE-S	LongSAGE-M	LongSAGE-S	Affy-M	Affy-S	M-S
Pearson	0.623	0.626	0.656	0.737	0.481	0.672	0.243	0.335	0.852
*P* value	0.004	0.004	0.002	<0.001	0.037	0.002	0.317	0.161	<0.001
Spearman	0.663	0.596	0.515	0.502	0.374	0.567	0.521	0.583	0.841
*P* value	0.002	0.008	0.024	0.03	0.115	0.013	0.022	0.009	<0.001

M: average expression stability calculated by the geNorm program, S: stability value calculated by the NormFinder program. For 48 samples, 20 reference genes including 13 novel genes and 7 classical genes, were used in the correlation analysis. For 60 FFPE tissues, 19 reference genes excluding DIMT1L were included in the analysis.

Consistently with frozen tissues and cell lines, both gene expression stability values demonstrated that most nERGs with low stability values are superior to tERGs in terms of expression stability in FFPE tissues (Table 5b), proving the usefulness of our nERGs as reliable measurements of gene expression in those tissues. *GPBP1* and *PAPOLA* were the two least variable genes in geNorm and *ARL8B* and *LUC7L2* were the top two ranked genes in NormFinder. However, in the analysis by each tissue, *FBXW2, TRIM27*, and *CUL1* showed high stability values in breast, ovary, and stomach, respectively (Table 7), suggesting that they have high expression variation in each tissue. Also, S values by NormFinder in the ovary and stomach FFPE tissues were calculated based on the combination of intra- and inter-group variations between normal and tumor samples. The relatively high S values of *TRIM27* in the ovary and *CUL1* in the stomach suggest that their expression might be regulated in specific tumors compared to their normal tissues.

**Table 7:** nERGs and tERGs ranked according to their expression stability, as calculated by the two programs, geNorm and NormFinder, based on qRT-PCR data in each tissue type of FFPE tissues.

Breast FFPE (n = 10)				Ovary FFPE (n = 33)*				Stomach FFPE (n = 17)*			
GeNorm		NormFinder		GeNorm		NormFinder*		GeNorm		NormFinder*	
Gene Symbol	Average expression stability M	Gene Symbol	Stability value S	Gene Symbol	Average expression stability M	Gene Symbol	Stability value S	Gene Symbol	Average expression stability M	Gene Symbol	Stability value S
TBP	0.186	LUC7L2	0.080	ARL8B	0.336	ARL8B	0.050	CTBP1	0.296	OAZ1	0.097
CTBP1		UBQLN1	0.138	FBXW2		SPG21	0.072	TRIM27		HMBS	0.123
PAPOLA	0.199	CTBP1	0.152	CTBP1	0.361	UBQLN1	0.091	FBXW2	0.325	FBXW2	0.162
OAZ1	0.231	CUL1	0.153	SPG21	0.390	CTBP1	0.110	LUC7L2	0.354	PAPOLA	0.171
CUL1	0.245	TBP	0.173	UBQLN1	0.409	FBXW2	0.114	OAZ1	0.387	TRIM27	0.200
ARL8B	0.265	SPG21	0.190	LUC7L2	0.417	HPRT1	0.117	HMBS	0.406	B2M	0.202
GPBP1	0.282	B2M	0.212	OAZ1	0.436	TBP	0.129	GPBP1	0.430	GAPDH	0.207
UBQLN1	0.298	OAZ1	0.216	PAPOLA	0.464	LUC7L2	0.131	PAPOLA	0.446	LUC7L2	0.216
LUC7L2	0.307	ARL8B	0.219	GPBP1	0.482	CUL1	0.165	ARL8B	0.464	ARL8B	0.226
ZNF207	0.317	GPBP1	0.222	CUL1	0.498	OAZ1	0.177	TBP	0.483	ZNF207	0.230
B2M	0.335	HMBS	0.240	TBP	0.515	GPBP1	0.184	ZNF207	0.512	ACTB	0.266
SPG21	0.356	PAPOLA	0.246	ZNF207	0.535	PPIA	0.212	UBQLN1	0.533	PPIA	0.275
HMBS	0.374	TRIM27	0.253	HPRT1	0.556	PAPOLA	0.248	GAPDH	0.556	UBQLN1	0.285
TRIM27	0.389	ZNF207	0.305	TRIM27	0.572	ZNF207	0.253	SPG21	0.575	CTBP1	0.288
PPIA	0.417	ACTB	0.316	HMBS	0.590	B2M	0.285	HPRT1	0.592	SPG21	0.293
ACTB	0.438	PPIA	0.317	PPIA	0.620	HMBS	0.323	B2M	0.614	GPBP1	0.298
HPRT1	0.464	HPRT1	0.392	B2M	0.645	TRIM27	0.326	PPIA	0.635	TBP	0.336
FBXW2	0.489	FBXW2	0.447	GAPDH	0.674	ACTB	0.466	ACTB	0.657	CUL1	0.342
GAPDH	0.530	GAPDH	0.570	ACTB	0.705	GAPDH	0.496	CUL1	0.682	HPRT1	0.354
				Best combination of two genes		ARL8B and SPG21	0.043	Best combination of two genes		CTBP1 and UBQLN1	0.066

* Gene expression stability S by NormFinder was calculated as an estimate of the combined intra and intergroup variation between normal and tumor tissues. For ovary tissues (n = 33), 10 normal and 23 tumor tissues were included and 17 stomach tissues, including normal (n = 8) and tumor (n = 9) tissues, were used in the analysis.

Low average expression stability M and stability value S indicate the high expression stability.

**Figure 5:**
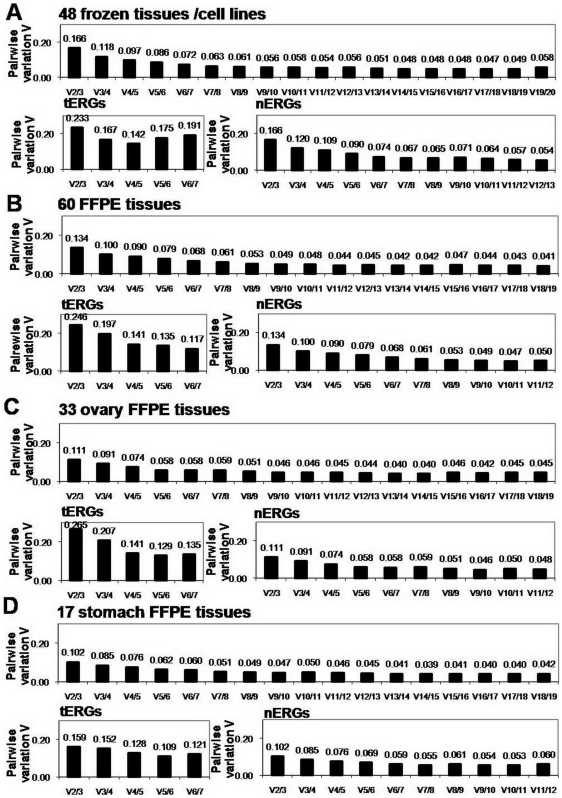
Optimal number of ERGs for normalization calculated by the geNorm program. Variable V defines the pair-wise variation between two sequential normalization factors containing an increasing number of genes. For example, V2/3 indicates the variation of the normalization factor of two genes in relation to three genes. A large V indicates that the added gene should be included for calculation of the normalization factor. 0.15 was proposed as a cut-off value, below which the inclusion of an additional reference gene is not required. Pair-wise variation analysis to determine the number of ERGs required for accurate normalization was performed in 48 samples including human frozen tissues and cell lines (A), 60 FFPE tissues (B), 33 ovary FFPE tissues (C) and 17 stomach FFPE tissues (D). For each case, the analysis was done for total ERGs, tERGs and nERGs.

The optimal number of ERGs for normalization was determined using geNorm. In both the 48 human frozen and cell line samples and 60 FFPE tissues, the optimal number of nERGs required for normalization was fewer than when using tERGs (Figure 5). Four tERGs and three nERGs were calculated as the optimal number of ERGs needed in the 48 samples when using a V of 0.15 as the cut-off value [1]. In the FFPE samples, V2/3 was under 0.15 when using nERGs, suggesting that only two genes are sufficient for optimal normalization, whereas four of seven tERGs were necessary for accurate normalization. This indicates that fewer ERGs are required for optimal normalization when using our nERGs rather than using tERGs.

## Discussion

In the present study, we identified nERGs in human samples using a comparative analysis of different large datasets of human gene expression profiles, while previous attempts to identify nERGs that are superior to tERGs were limited to the analysis of a microarray [10], [18]–[21] or EST data [35].

Candidate HKGs were first selected and included 2,087 genes, which is a larger number of genes than previously identified by other groups [27], [32], [36]. Their characteristics, including high levels of expression [27], [36] and the prevalence of CpG islands in the promoter regions [33], [34], were in line with previous studies based on smaller numbers of HKGs, reflecting that “real” HKGs showing constitutive expressions in all tissues are enriched in our list. Thus, this list can be used as a reliable source for the study of HKGs.

The 13 nERGs further identified from candidate HKGs showed relatively lower CV and lower expression than most of the tERGs in all datasets. These findings were further confirmed by qRT-PCR using frozen tissues and cell lines. Generally, the expression of 13 nERGs was lower than the highly expressed tERGs and higher or similar to the weakly expressed tERGs. The expression stability values of the nERGs calculated by both programs also demonstrated that nERGs are generally more stably expressed than tERGs. Although there were slight differences in their rankings between the two programs, *PAPOLA, CUL1, TBP, LUC7L2, GPBP1* and *TRIM27* were found to be the genes with the most stable expression in 48 samples. The observation that *TBP* is one of the most stable genes is not surprising because relatively lower variability of TBP among traditional ERGs was already expected in datasets including EST and ShortSAGE (Figure 3). On the other hand, our data further supported the unsuitability of the most commonly used ERGs, like *GAPDH, ACTB,* for normalization, in line with previous works [7], [8], [14].

Our nERGs were also successfully validated by qRT-PCR in FFPE tissues. Despite the usefulness of archival FFPE tissue specimens in conjunction with clinical data, frequent degradation of RNA from FFPE tissues has been regarded as an obstacle in the gene expression analysis of those samples [37]. The expression of nERGs was measurable with reliable Cp values in all 60 FFPE tissues and most of the nERGs outperformed tERGs with respect to expression stability.

Furthermore, the expression level of target gene can be calculated relative to the expression level of one or multiple nERGs using standard curve or comparative Ct(Cp) method in quantitative gene expression analyses, including qRT-PCR. The most suitable ERG or ERGs in the designed experiment can be selected among 13 nERGs or combination of tERGs and 13 nERGs based on gene expression stability values calculated by the geNorm and/or NormFinder program. Recently developed PCR array, high throughput gene expression measurement using qRT-PCR, also requires more suitable ERGs than conventional tERGs for accurate quantification of gene expression [38]. Recently, normalization using the geometric mean of multiple ERGs has been considered to be more accurate for normalization [1], especially in situations when no optimal ERG has been identified [31]. Moreover, the use of multiple ERGs is more important in the expression analysis using FFPE tissues, where poor RNA quality causes additional variations in gene expression [39]. Five tERGs are included for the normalization of gene expression in the Onco*type* DX™ assay (Genomic Health) which is a clinical validated multigene qRT-PCR test using FFPE tissues to predict recurrence of breast cancer [40]. However, the superiority of our nERGs was demonstrated by showing that a fewer number of genes is required for normalization when using the nERGs as compared with using the tERGs. Considering the limited amounts of samples or experiment cost, nERGs also outperformed previous tERGs when multiple genes are needed for reliable normalization. Taken together, nERGs will be a better choice for more reliable normalization in gene expression analysis using FFPE tissues.

The superiority of nERGs over tERGs is based in their lower expression level as well as their high expression stability. Use of ERGs with expression levels similar to target genes is recommended so that the comparisons fall on the same linear scale [19], [41], [42]. Therefore, our nERGs, with relatively lower expression, rather than *GAPDH* or *ACTB* showing high expression, can be better candidates for normalization of a wide range of genes, including weakly expressed genes. This is significant given that the majority of transcripts in human tissues are expressed in low abundance [36].

Remarkably, we observed no significant correlation between the stability values calculated from qRT-PCR data and CV in the Affymetrix data, which is in contrast to the significant correlation between stability values and CV in the EST and SAGE dataset. This suggests that EST or shortSAGE may be better sources for exploring nERGs rather than microarrays, which have traditionally been used as a source for screening ERG and supports our initial hypothesis that the microarray might not be a good source for ERGs.

Furthermore, nERGs identified here might be used as reference for relative measurements of gene amplification, which is a frequent genetic alteration leading to unregulated gene expression in cancer [43]. As the relatively constant expression of these genes in both normal and tumor tissues provides the possibility that these genes are located in a chromosomal region in which no genetic alterations are found in human tumors, we investigated the genomic CNVs of nERGs using publicly available databases. Most nERGs, except *OAZ1* and *DIMT1L,* were located in genomic regions where CNVs were not reported, whereas many tERGs were located in regions with CNVs. The relatively lower expression stability of *OAZ1* and *DIMT1L*, as well as tERGs like *GAPDH* and *ACTB*, might be explained by these genomic aberrations. However, the suitability of nERGs as a reference for the relative measurement of gene amplification remains to be further investigated and validated through experiments. Meanwhile, even genes with genomic variations can be used for the normalization in gene expression, provided that their expression is not affected by genomic aberrations.

In conclusion, we have identified a set of candidate HKGs and nERGs based on a comparative analysis of EST, SAGE, and Affymetrix datasets. This is the first study using three different platforms to identify nERGs, and most of the 13 ERGs identified in these datasets were demonstrated to be more stably expressed than tERGs through the validation using qRT-PCR in large human samples including cell lines, frozen tissues and FFPE tissues. Moreover, these nERGs were expressed at relatively lower levels than most commonly used high expressing tERGs, making them more suitable for normalization of transcripts from a wide range of expression levels. We have also shown that fewer ERGs are required for accurate normalization using nERGs than using tERGs, especially in FFPE tissues when the use of multiple ERGs is required.

## Supporting Information

Text S1 Supplementary materials and methods
(0.09 MB DOC)

Text S2 Expression of 2,087 candidate HKGs in the four datasets
(0.04 MB DOC)

Table S1 List of 567 samples including 13 tissue types in the HG-U133 array used in this study
(0.07 MB DOC)

Table S2 A list of 2,087 candidate housekeeping genes
(0.86 MB XLS)

Table S3 Human frozen tissues and cancer cell lines used in qRT-PCR
(0.08 MB DOC)

Table S4 Correlation of gene expression and CV of 2,087 candidate HKGs between the four datasets
(0.05 MB DOC)

Table S5 CpG islands analysis in the upstream region from transcription start site of 13 nERGs
(0.04 MB DOC)

Table S6 Correlation of gene expression and CV of 13 nERGs between the four datasets
(0.05 MB DOC)

Table S7 tERGs used in this study
(0.05 MB DOC)

Table S8 Comparison of CV between nERGs and tERGs in the dataset
(0.04 MB DOC)

Table S9 Comparison of Cp values between nERGs and tERGs in qRT-PCR
(0.04 MB DOC)

Table S10 Comparison of gene expression stability values between nERGs and tERGs
(0.04 MB DOC)

## References

[1] Vandesompele J, De Preter K, Pattyn F, Poppe B, Van Roy N (2002). Accurate normalization of real-time quantitative RT-PCR data by geometric averaging of multiple internal control genes.. Genome Biol.

[2] Thellin O, Zorzi W, Lakaye B, De Borman B, Coumans B (1999). Housekeeping genes as internal standards: use and limits.. J Biotechnol.

[3] Bustin SA (2000). Absolute quantification of mRNA using real-time reverse transcription polymerase chain reaction assays.. J Mol Endocrinol.

[4] Tricarico C, Pinzani P, Bianchi S, Paglierani M, Distante V (2002). Quantitative real-time reverse transcription polymerase chain reaction: normalization to rRNA or single housekeeping genes is inappropriate for human tissue biopsies.. Anal Biochem.

[5] Rubie C, Kempf K, Hans J, Su T, Tilton B (2005). Housekeeping gene variability in normal and cancerous colorectal, pancreatic, esophageal, gastric and hepatic tissues.. Mol Cell Probes.

[6] Schmittgen TD, Zakrajsek BA (2000). Effect of experimental treatment on housekeeping gene expression: validation by real-time, quantitative RT-PCR.. J Biochem Biophys Methods.

[7] Zhong H, Simons JW (1999). Direct comparison of GAPDH, beta-actin, cyclophilin, and 28S rRNA as internal standards for quantifying RNA levels under hypoxia.. Biochem Biophys Res Commun.

[8] Selvey S, Thompson EW, Matthaei K, Lea RA, Irving MG (2001). Beta-actin–an unsuitable internal control for RT-PCR.. Mol Cell Probes.

[9] Lee PD, Sladek R, Greenwood CM, Hudson TJ (2002). Control genes and variability: absence of ubiquitous reference transcripts in diverse mammalian expression studies.. Genome Res.

[10] Hamalainen HK, Tubman JC, Vikman S, Kyrola T, Ylikoski E (2001). Identification and validation of endogenous reference genes for expression profiling of T helper cell differentiation by quantitative real-time RT-PCR.. Anal Biochem.

[11] Dheda K, Huggett JF, Chang JS, Kim LU, Bustin SA (2005). The implications of using an inappropriate reference gene for real-time reverse transcription PCR data normalization.. Anal Biochem.

[12] de Kok JB, Roelofs RW, Giesendorf BA, Pennings JL, Waas ET (2005). Normalization of gene expression measurements in tumor tissues: comparison of 13 endogenous control genes.. Lab Invest.

[13] Huggett J, Dheda K, Bustin S, Zumla A (2005). Real-time RT-PCR normalisation; strategies and considerations.. Genes Immun.

[14] Goidin D, Mamessier A, Staquet MJ, Schmitt D, Berthier-Vergnes O (2001). Ribosomal 18S RNA prevails over glyceraldehyde-3-phosphate dehydrogenase and beta-actin genes as internal standard for quantitative comparison of mRNA levels in invasive and noninvasive human melanoma cell subpopulations.. Anal Biochem.

[15] Haller F, Kulle B, Schwager S, Gunawan B, von Heydebreck A (2004). Equivalence test in quantitative reverse transcription polymerase chain reaction: confirmation of reference genes suitable for normalization.. Anal Biochem.

[16] Ohl F, Jung M, Xu C, Stephan C, Rabien A (2005). Gene expression studies in prostate cancer tissue: which reference gene should be selected for normalization?. J Mol Med.

[17] Radonic A, Thulke S, Mackay IM, Landt O, Siegert W (2004). Guideline to reference gene selection for quantitative real-time PCR.. Biochem Biophys Res Commun.

[18] Hoerndli FJ, Toigo M, Schild A, Gotz J, Day PJ (2004). Reference genes identified in SH-SY5Y cells using custom-made gene arrays with validation by quantitative polymerase chain reaction.. Anal Biochem.

[19] Czechowski T, Stitt M, Altmann T, Udvardi MK, Scheible WR (2005). Genome-wide identification and testing of superior reference genes for transcript normalization in Arabidopsis.. Plant Physiol.

[20] Jin P, Zhao Y, Ngalame Y, Panelli MC, Nagorsen D (2004). Selection and validation of endogenous reference genes using a high throughput approach.. BMC Genomics.

[21] Shulzhenko N, Yambartsev A, Goncalves-Primo A, Gerbase-DeLima M, Morgun A (2005). Selection of control genes for quantitative RT-PCR based on microarray data.. Biochem Biophys Res Commun.

[22] Lipshutz RJ, Fodor SP, Gingeras TR, Lockhart DJ (1999). High density synthetic oligonucleotide arrays.. Nat Genet.

[23] Haverty PM, Hsiao LL, Gullans SR, Hansen U, Weng Z (2004). Limited agreement among three global gene expression methods highlights the requirement for non-global validation.. Bioinformatics.

[24] van Ruissen F, Ruijter JM, Schaaf GJ, Asgharnegad L, Zwijnenburg DA (2005). Evaluation of the similarity of gene expression data estimated with SAGE and Affymetrix GeneChips.. BMC Genomics.

[25] Sun M, Zhou G, Lee S, Chen J, Shi RZ (2004). SAGE is far more sensitive than EST for detecting low-abundance transcripts.. BMC Genomics.

[26] Lee S, Jo M, Lee J, Koh SS, Kim S (2007). Identification of novel universal housekeeping genes by statistical analysis of microarray data.. J Biochem Mol Biol.

[27] Eisenberg E, Levanon EY (2003). Human housekeeping genes are compact.. Trends Genet.

[28] Ruepp A, Zollner A, Maier D, Albermann K, Hani J (2004). The FunCat, a functional annotation scheme for systematic classification of proteins from whole genomes.. Nucleic Acids Res.

[29] Wang Y, Leung FC (2004). An evaluation of new criteria for CpG islands in the human genome as gene markers.. Bioinformatics.

[30] Ramakers C, Ruijter JM, Deprez RH, Moorman AF (2003). Assumption-free analysis of quantitative real-time polymerase chain reaction (PCR) data.. Neurosci Lett.

[31] Andersen CL, Jensen JL, Orntoft TF (2004). Normalization of real-time quantitative reverse transcription-PCR data: a model-based variance estimation approach to identify genes suited for normalization, applied to bladder and colon cancer data sets.. Cancer Res.

[32] Hsiao LL, Dangond F, Yoshida T, Hong R, Jensen RV (2001). A compendium of gene expression in normal human tissues.. Physiol Genomics.

[33] Larsen F, Gundersen G, Lopez R, Prydz H (1992). CpG islands as gene markers in the human genome.. Genomics.

[34] Ponger L, Duret L, Mouchiroud D (2001). Determinants of CpG islands: expression in early embryo and isochore structure.. Genome Res.

[35] Coker JS, Davies E (2003). Selection of candidate housekeeping controls in tomato plants using EST data.. Biotechniques.

[36] Warrington JA, Nair A, Mahadevappa M, Tsyganskaya M (2000). Comparison of human adult and fetal expression and identification of 535 housekeeping/maintenance genes.. Physiol Genomics.

[37] Bibikova M, Talantov D, Chudin E, Yeakley JM, Chen J (2004). Quantitative gene expression profiling in formalin-fixed, paraffin-embedded tissues using universal bead arrays.. Am J Pathol.

[38] Spurgeon SL, Jones RC, Ramakrishnan R (2008). High throughput gene expression measurement with real time PCR in a microfluidic dynamic array.. PLoS ONE.

[39] Glenn ST, Jones CA, Liang P, Kaushik D, Gross KW (2007). Expression profiling of archival renal tumors by quantitative PCR to validate prognostic markers.. Biotechniques.

[40] Paik S, Shak S, Tang G, Kim C, Baker J (2004). A multigene assay to predict recurrence of tamoxifen-treated, node-negative breast cancer.. N Engl J Med.

[41] Szabo A, Perou CM, Karaca M, Perreard L, Quackenbush JF (2004). Statistical modeling for selecting housekeeper genes.. Genome Biol.

[42] RocheAppliedScience (2005). Technical Note No. LC 15/2005..

[43] Lehmann U, Glockner S, Kleeberger W, von Wasielewski HF, Kreipe H (2000). Detection of gene amplification in archival breast cancer specimens by laser-assisted microdissection and quantitative real-time polymerase chain reaction.. Am J Pathol.

