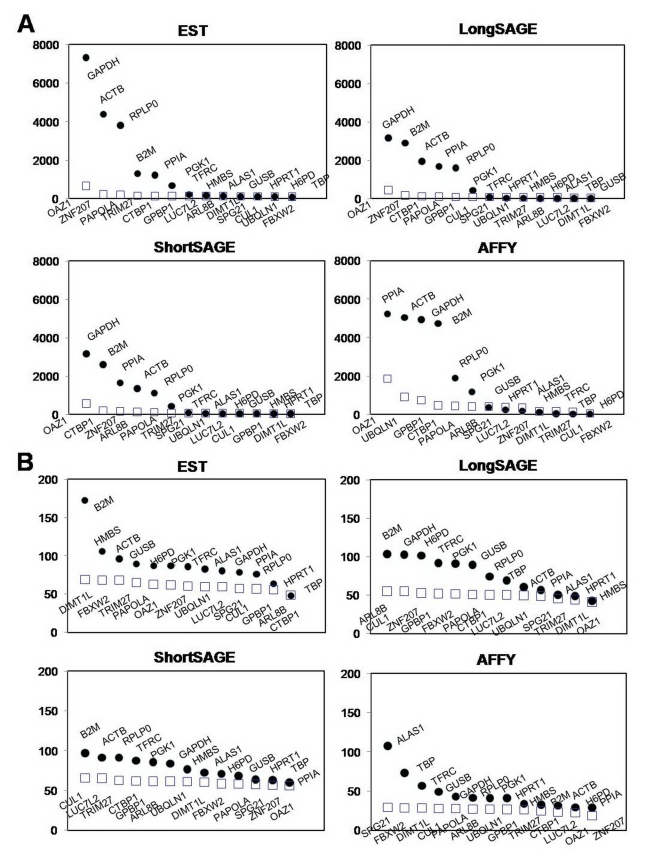# Correction: Identification of Novel Reference Genes Using Multiplatform Expression Data and Their Validation for Quantitative Gene Expression Analysis

**DOI:** 10.1371/annotation/695436c7-3329-4bdc-9832-f427ecc33698

**Published:** 2009-08-07

**Authors:** Mi Jeong Kwon, Ensel Oh, Seungmook Lee, Mi Ra Roh, Si Eun Kim, Yangsoon Lee, Yoon-La Choi, Yong-Ho In, Taesung Park, Sang Seok Koh, Young Kee Shin

Figure 3 was incorrect as published. Please see the corrected figure here: 

**Figure:**