# Supplementary material for: Identification of Novel Reference Genes Using Multiplatform Expression Data and Their Validation for Quantitative Gene Expression Analysis
Source: PLoS One. 2009 Jul 7;4(7):e6162. doi: 10.1371/journal.pone.0006162 (PMC2703796; doi:10.1371/journal.pone.0006162)
Supplement: Text S1 — Supplementary materials and methods (0.09 MB DOC) [file pone.0006162.s001.doc]

**Supplementary Materials and Methods**

**EST, SAGE, and microarray gene expression dataset construction**

*EST dataset*

EST human gene expression data were collected from the publicly available Cancer Genome Anatomy Project (CGAP) site (http://cgap.nci.nih.gov/). Among a total of 8,633 libraries in the Hs_LibData.dat (31/Oct/05) file, we identified 77 libraries which met the necessary requirements for constructing the EST gene expression dataset. Specifically, the libraries were non-normalized and contained >10,000 sequences. The frequencies of specific ESTs in each library were obtained from the Hs_ExprData.dat (31/Oct/05) file. A total of 29 different tissues, including normal and tumor samples, and 26,117 UniGene clusters were included in these 77 libraries.

*Short and long SAGE datasets*

SAGE human gene expression data were also collected from the publicly available CGAP site (http://cgap.nci.nih.gov/). We obtained short SAGE tag (10bp) data from all 280 libraries in the Hs_short.frequencies.gz file (05/Dec/06) including 38,290 UniGene clusters and long SAGE tag (17bp) data from 46 libraries in the Hs_long.frequencies.gz file (05/Dec/06) including 21,868 UniGene clusters, respectively and these data were used in creating the short and long SAGE datasets. SAGE tags were mapped to UniGene clusters using both the Hs_short.best_gene.gz (05/Dec/06) and Hs_long.best_gene.gz (05/Dec/06) files. The short and long SAGE datasets are composed of 28 and 9 different tissues including normal and tumor tissues, respectively.

*Microarray dataset*

Microarray gene expression data were obtained from the Cancer Genome Expression Database of LG Life Sciences, Ltd., which integrates the GeneExpress Oncology Datasuite^TM^ of Gene Logic Inc., and LG's Proprietary Cancer Genome Database based on the Affymetrix Human Genome U133 (HG-U133) array set.

Affymetrix GeneChip gene expression data from 567 different samples representing 13 different tissues including brain, breast, colon, esophagus, kidney, liver, lung, lymph node, ovary, pancreas, prostate, rectum, and stomach were analyzed (Table S1). Probes for 44,760 different genes (a total of 44,928 probe sets) were included in the Affymetrix data.

**Calculating expression level values in EST, SAGE and Affymetrix GeneChip datasets**

We calculated the EST gene expression level as the number of ESTs of a gene in a given library, divided by the total number of ESTs in the library and then multiplied by 1,000,000　[1]．SAGE gene expression levels for each gene were calculated as the number of tags for a gene in a given library, divided by total number of tags in the library and then multiplied by 1,000,000 [1]. Thus, Gene A’s expression from library B is defined as follows for EST and SAGE [1]:

$$\text{EST gene expression }=\text{ }\frac{\text{No. of gene A ESTs in library B}}{\text{Total No. of ESTs in library B }}\times1,000,000$$

$$\text{SAGE gene expression }=\text{ }\frac{\text{No. of gene A SAGE tags in library B}}{\text{Total No. of SAGE tags in library B }}\times1,000,000$$

Affymetrix GeneChip expression levels were defined by the signal intensity for each transcript, as calculated by the analysis algorithm in the Affymetrix Microarray Suite 5.0 software. Mean gene expression was calculated as the average of the gene expression levels of a given gene in all libraries.

**Determination of cut-off values for 0’s proportion in each dataset for the identification of candidate HKGs**

Genes with 0’s proportion <0.4 for ESTs (4,027 UniGene clusters), <0.1 for short SAGE (4,758 UniGene clusters), and <0.3 for long SAGE (4,569 UniGene clusters) were selected, respectively and of these genes meeting above criteria in each dataset, 2,087 genes (Table S2) common to three datasets were identified and categorized as candidate HKGs in our datasets. Cut-off values for 0’s proportion in each dataset were set to include most of the 575 HKGs identified from previous Affymetrix microarray data using the U95A chip representing 12,600 probes in 101 different samples from 47 different human tissues and cell lines [2]. Of these 575 genes, 451 (78.43%) genes were included in the EST dataset, 432 (75.13%) in the short SAGE dataset, and 446 (77.57%) in the long SAGE dataset. The majority of the 575 HKGs were observed to have low 0’s proportion in our EST, short and long SAGE datasets, although some genes had an 0’s proportion greater than 0.5. Affymetrix data for 5,317 probe sets (5,238 different probe sets, 1,961 UniGene clusters) corresponding to 2087 HKGs were obtained.

**Functional classification of candidate HKGs and CpG island analysis of their putative promoters**

Candidate HKGs identified in this study were classified using the Functional Classification Catalogue (FunCat, Version 2.0), a system which classifies proteins according to cellular function [3]. The Hs.GeneData.dat file(31/Oct/05, ftp://ftp1.nci.nih.gov/pub/CGAP/

HsGeneData.dat), gene_ontology.obo file (format version1.0, http://www.geneontology.org/

ontology/gene_ontology.obo) and mips2go.txt file (http://www.geneontology.org/external2go

/mips2go) were used for classifying each candidate HKGs by FunCat. Gene ontology (GO) terms for candidate HKGs were mapped to the MIPS Functional Catalogue using the mips2go.txt file. Of the 2,087 candidate HKGs, 1,689 UniGene clusters were mapped and 398 UniGene clusters were excluded due to absence of linking between the UniGene cluster, GO and FunCat classifications. Among the 1,689 UniGene clusters, 1,318 UniGene clusters with FunCat classifications corresponding to GO terms for biological processes were identified.

For CpG island analysis, sequences upstream of the annotated transcription start site of RefSeq genes were obtained from the UCSC site (<http://hgdownload.cse.ucsc.edu/goldenPath/hg18/bigZips/>). CpG islands in sequences 1,000, 2,000, and 5,000 bases upstream of the transcription start site were searched using the CpGIE software [4], using the following criteria: a DNA sequence longer than 500 bp with a G+C content ≥55% and a CpG obs/exp (o/e) ratio ≥0.65 [5].

**Total RNA and cDNA preparation from human frozen, formalin-fixed paraffin-embedded (FFPE) tissues and human cancer cell lines and qRT-PCR**

First-strand cDNA for frozen tissues and cancer cell lines was synthesized from 2 μg of total RNA using the Superscript II First strand synthesis system (Invitrogen Life Technologies) according to the manufacturer’s protocol. The cDNA was then diluted 1:3 for use in qRT-PCR. RT was confirmed by PCR using primers designed to amplify a fragment of the beta actin (*ACTB*) gene.

For total RNA extraction from FFPE tissues, three 8 μm thick sections were cut from each paraffin block. Each tissue block was stained with hematoxylin and normal or tissue regions were microdissected using Laser Microdissection System (ION LMD, JungWoo International, Korea) or cut directly with needle from section. Paradise Whole Transcript RT Reagent System (Arcturus, CA, USA) was used for RNA isolation and RT of FFPE samples. Due to the limitation of RNA amount extracted from FFPE tissues, half RNA and cDNA were used for reverse transcription and qRT-PCR, respectively.

Primers and Taqman probes were designed using Probe Finder in the Universal Probe Library (UPL) Assay Design Center (Roche Applied Science, Mannheim, Germany). The primers and Taqman probes used for the study are shown in Table 1. tERGs for qRT-PCR were chosen based on the frequency of their use in the literature and in commercial kits and CV expected in our datasets. The 8 most commonly used ERGs are included in commercial kits such as Taqman human endogenous control plate (Applied Biosystems) and the HKG selection kit (Roche Applied Science, Mannheim, Germany). Taqman probes were 5’ labeled with the fluorescent reporter dye 6-carboxy-fluorescein (FAM) and the quencher dye 6-carboxy-tetramethyl-rhodamine (TAMARA). Each gene was detected at 530 nm using a FAM-conjugated UPL probe (Roche Applied Science) or custom-made specific probe (TIB MOLBIOL GmbH, Berlin, Germany). All PCR reactions were performed in a Lightcycler 2.0 (Roche Applied Science). The reaction consisted of 2 μl of template cDNA, 1 μl of primer mix (10 pmol/μl), 1 μl of Taqman probe (1 pmol/μl) and 2 μl of Master Mix (Roche Applied Science) in a total volume of 10 μl. Amplifications were initiated by a 10 min pre-incubation at 95 ℃, followed by 45 cycles of 10 s at 95 ℃ for template denaturation and 30 s at 55 ℃ for primer annealing/extension and a cooling step for 30 s at 40 ℃. 1μl of template cDNA was used for FFPE tissue samples. For quantitative comparison of mRNA levels of the candidate reference genes and traditional reference genes, the “crossing point” (Cp) values were used. In the LightCycler software, Cp is synonymous with the “threshold cycle” (Ct) value and represents the point at which the fluorescence signal exceeds the background fluorescence. The Cp of *H6PD* was not able to be determined in four frozen tissue samples, including normal tissues of lung, liver, breast and kidney; thus, this gene was excluded from subsequent calculations. The Cp of *DIMT1L* was not detectable in six FFPE tissue samples including 1 normal stomach, 2 stomach carcinomas, 1 ovarian borderline tumor and 2 ovarian cancers, so excluded from gene expression stability analysis for FFPE samples.

**Determination of PCR efficiency in qRT-PCR experiments**

*Dilution Method*

PCR efficiency was determined for each gene by measuring the Cp values of serial dilutions (1, 1:4, 1:16, 1:64) of 1/2 diluted cDNA from MKN 74 cells, a gastric cancer cell line, to ensure comparability between all assays and to calculate the relative expression. PCR efficiencies were calculated from the LightCycler software 4.0 and are shown in Table 1.

*LinRegPCR program*

We also estimated PCR efficiency of each probe for each tissue sample with the LinRegPCR program, an alternative method using linear regression on the Log (fluorescence) per cycle data of each qRT-PCR reaction [6]. PCR efficiencies for all probes calculated using the LinRegPCR program are shown in Table 1. Each PCR efficiency represents an average ± SD calculated from amplification plots of 137-143 samples (triplicate samples of 48 different cDNAs), and 64 samples for *H6PD* (triplicate samples of 22 different cDNAs) [7].

**Gene expression stability analysis**

To compare the gene expression stability of candidate reference genes to traditional reference genes and to choose the most stable genes, two programs, geNorm and NormFinder, were used. The relative expression level was calculated as (1+E)^△Cp^, where △Cp = min Cp - sample Cp, minCp = lowest Cp value, and E = PCR efficiency [8].

The gene expression stability measure "M" in geNorm is calculated based on the average pairwise variation between all genes studied. The gene with the highest M value is the least stable gene and stepwise exclusion of the gene with the highest M value allows the ranking of the tested genes according to their expression stability and leads to the identification of the two most stable genes [9]. This program also calculates the optimal reference genes for accurate normalization. NormFinder calculates the stability value based on the combined estimate of intra and intergroup expression variations of the genes tested and a low stability value indicates high expression stability [10]. To investigate the relationship between the expression stability values and the CV values in each dataset for 20 reference genes (19 genes for 60FFPE samples), Pearson and Spearman correlation analysis was performed, respectively. The Pearson and Spearman correlation coefficients are a measure of the linear association between two groups. A *P* value <0.05 was considered significant. All calculations were produced using the R statistical software.

**References**

1. Haverty PM, Hsiao LL, Gullans SR, Hansen U, Weng Z (2004) Limited agreement among three global gene expression methods highlights the requirement for non-global validation. Bioinformatics 20: 3431-3441.

2. Eisenberg E, Levanon EY (2003) Human housekeeping genes are compact. Trends Genet 19: 362-365.

3. Ruepp A, Zollner A, Maier D, Albermann K, Hani J, et al. (2004) The FunCat, a functional annotation scheme for systematic classification of proteins from whole genomes. Nucleic Acids Res 32: 5539-5545.

4. Wang Y, Leung FC (2004) An evaluation of new criteria for CpG islands in the human genome as gene markers. Bioinformatics 20: 1170-1177.

5. Takai D, Jones PA (2002) Comprehensive analysis of CpG islands in human chromosomes 21 and 22. Proc Natl Acad Sci U S A 99: 3740-3745.

6. Ramakers C, Ruijter JM, Deprez RH, Moorman AF (2003) Assumption-free analysis of quantitative real-time polymerase chain reaction (PCR) data. Neurosci Lett 339: 62-66.

7. Czechowski T, Stitt M, Altmann T, Udvardi MK, Scheible WR (2005) Genome-wide identification and testing of superior reference genes for transcript normalization in Arabidopsis. Plant Physiol 139: 5-17.

8. Balch C, Nephew KP, Huang TH, Bapat SA (2007) Epigenetic "bivalently marked" process of cancer stem cell-driven tumorigenesis. Bioessays 29: 842-845.

9. Vandesompele J, De Preter K, Pattyn F, Poppe B, Van Roy N, et al. (2002) Accurate normalization of real-time quantitative RT-PCR data by geometric averaging of multiple internal control genes. Genome Biol 3: RESEARCH0034.

10. Andersen CL, Jensen JL, Orntoft TF (2004) Normalization of real-time quantitative reverse transcription-PCR data: a model-based variance estimation approach to identify genes suited for normalization, applied to bladder and colon cancer data sets. Cancer Res 64: 5245-5250.
