# Supplementary material for: Identification of Novel Reference Genes Using Multiplatform Expression Data and Their Validation for Quantitative Gene Expression Analysis
Source: PLoS One. 2009 Jul 7;4(7):e6162. doi: 10.1371/journal.pone.0006162 (PMC2703796; doi:10.1371/journal.pone.0006162)
Supplement: Text S2 — Expression of 2,087 candidate HKGs in the four datasets (0.04 MB DOC) [file pone.0006162.s002.doc]

**Supplementary Data**

**Expression of 2,087 candidate HKGs in the four datasets**

The expression of candidate HKGs ranged 73.83 to 9,324.87 in EST libraries, 28.58 to 4,086.25 in short SAGE libraries, 17.47 to 4,621.60 in long SAGE libraries, and 1.47 to 18,598.26 in the HG-U133 microarray (Table S2). Non-HKGs showed the expression range of 20.94 to 18,344.22 for EST, 2.49 to 5,957.41 for short SAGE, 2.49 to 23,515.27 for long SAGE and 0.84 to 19,821.42 for Affymetrix, respectively (data not shown). Many ribosomal proteins were included in the most highly expressed candidate HKGs in all four datasets. *EEF1A1* was the most abundant transcript in both EST and long SAGE datasets (Table S2). *DGKI* and *RPL10A* were the most highly expressed genes in the short SAGE dataset and in the HG-U133 microarray, respectively (Table S2).
