# Supplementary material for: Identification of Novel Reference Genes Using Multiplatform Expression Data and Their Validation for Quantitative Gene Expression Analysis
Source: PLoS One. 2009 Jul 7;4(7):e6162. doi: 10.1371/journal.pone.0006162 (PMC2703796; doi:10.1371/journal.pone.0006162)
Supplement: Table S1 — List of 567 samples including 13 tissue types in the HG-U133 array used in this study (0.07 MB DOC) [file pone.0006162.s003.doc]

**Table S1.** List of 567 samples including 13 tissue types in the HG-U133 array used in this study

| **Tissue** | **Category** | **Morphology** | **Number of samples** | |
| --- | --- | --- | --- | --- |
| Brain | Benign | Meningioma | 7 | 23 |
|  | Malignant | Glioblastoma Multiforme | 7 |  |
|  |  | Oligodendroglioma | 6 |  |
|  |  | Medulloblastoma | 3 |  |
| Breast | Normal | Normal Tissue | 18 | 74 |
|  | Malignant | Infiltrating Duct Carcionma | 36 |  |
|  |  | Infiltrating Duct and Lobular Carcinoma | 7 |  |
|  |  | Infiltrating Lobular Carcinoma | 13 |  |
| Colon | Normal | Normal Tissue | 22 | 62 |
|  | Malignant | Adenocarcinoma | 33 |  |
|  |  | Mucinous Adenocarcinoma | 7 |  |
| Esophagus | Normal | Normal Tissue | 11 | 17 |
|  | Malignant | Adenocarcinoma | 6 |  |
| Kidney | Normal | Normal Tissue | 26 | 51 |
|  | Benign | Oncocytoma | 5 |  |
|  | Malignant | Clear Cell Adenocarcinoma | 6 |  |
|  |  | Renal Cell Carcinoma | 14 |  |
| Liver | Normal | Normal Tissue | 10 | 29 |
|  | Malignant | Hepatocellular Carcinoma | 19 |  |
| Lung | Normal | Normal Tissue | 26 | 58 |
|  | Malignant | Adenocarcinoma | 15 |  |
|  |  | Squamous Cell Carcinoma | 17 |  |
| Lymph Node | Normal | Normal Tissue | 4 | 23 |
|  | Malignant | Hodgkin’s Disease | 4 |  |
|  |  | Malignant Lymphoma | 15 |  |
| Ovary | Normal | Normal Tissue | 10 | 50 |
|  | Malignant | Adenocarcinoma | 5 |  |
|  |  | Clear Cell Adenocarcinoma | 6 |  |
|  |  | Mucinous Cystadenocarcinoma | 5 |  |
|  |  | Serous Cystadenocarcinoma | 6 |  |
|  |  | Papillary Serous Adenocarcinoma | 18 |  |
| Pancreas | Normal | Normal Tissue | 13 | 41 |
|  | Malignant | Adenocarcinoma | 28 |  |
| Prostate | Normal | Normal Tissue | 14 | 41 |
|  | Malignant | Adenocarcinoma | 27 |  |
| Rectum | Normal | Normal Tissue | 17 | 38 |
|  | Malignant | Adenocarcinoma | 21 |  |
| Stomach | Normal | Normal Tissue | 17 | 60 |
|  | Malignant | Adenocarcinoma | 37 |  |
|  |  | Signet Ring Cell Carcinoma | 6 |  |
| Total | | | 567 | |
