# Supplementary material for: Identification of Novel Reference Genes Using Multiplatform Expression Data and Their Validation for Quantitative Gene Expression Analysis
Source: PLoS One. 2009 Jul 7;4(7):e6162. doi: 10.1371/journal.pone.0006162 (PMC2703796; doi:10.1371/journal.pone.0006162)
Supplement: Table S3 — Human frozen tissues and cancer cell lines used in qRT-PCR (0.08 MB DOC) [file pone.0006162.s005.doc]

**Table S3.** Human frozen tissues and cancer cell lines used in qRT-PCR

| **No** | **Tissue or cell lines** | **Type** | **Normal(N)/Tumor(T)** | **Diagnosis** | **Remarks** |
| --- | --- | --- | --- | --- | --- |
| 1 | Adrenal gland | frozen tissue | N | Normal |  |
| 2 | Brain | frozen tissue | N | Normal |  |
| 3 | Breast | frozen tissue | N | Normal |  |
| 4 | Colon | frozen tissue | N | Normal | Normal tissue adjacent to signet ring cell carcinoma, ascending colon |
| 5 | Esophagus | frozen tissue | N | Normal |  |
| 6 | Kidney | frozen tissue | N | Normal |  |
| 7 | Liver | frozen tissue | N | Normal |  |
| 8 | Lung | frozen tissue | N | Normal |  |
| 9 | Omentum | frozen tissue | N | Normal |  |
| 10 | Ovary | frozen tissue | N | Normal |  |
| 11 | Placenta | frozen tissue | N | Normal |  |
| 12 | Placenta | frozen tissue | N | Normal | Immature placenta with focal intervillous calcifications, normal two umbilical arteries and one vein, and no evidence of chorioamnionitis |
| 13 | Rectum | frozen tissue | N | Normal |  |
| 14 | Salivary gland | frozen tissue | N | Normal |  |
| 15 | Thyroid gland | frozen tissue | N | Normal |  |
| 16 | Tonsil | frozen tissue | N | Normal |  |
| 17 | Uterus | frozen tissue | N | Normal |  |
| 18 | Vein | frozen tissue | N | Normal |  |
| 19 | Vulva | frozen tissue | N | Normal |  |
| 20 | Brain | frozen tissue | T | Glioblastoma mulfiforme |  |
| 21 | Breast | frozen tissue | T | Invasive ductal carcinoma, upper central |  |
| 22 | Transverse colon | frozen tissue | T | Ulcerofungating carcinoma, transverse colon, Mucinous adenocarcinoma |  |
| 23 | Lung | frozen tissue | T | Pleomorphic carcinoma |  |
| 24 | Ovary | frozen tissue | T | Transitional cell carcinoma, bilateral ovaries |  |
| 25 | Rectum | frozen tissue | T | Adenocarcinoma, moderately differentiated with mucin production |  |
| 26 | Stomach | frozen tissue | T | Advanced gastric carcinoma, Tubular adenocarcinoma, M/D |  |
| 27 | HL-60 | cell lines | T | Leukemia | Blood |
| 28 | MDA-MB-231 | cell lines | T | Breast cancer | Breast |
| 29 | C33A | cell lines | T | Cervical cancer | Cervix |
| 30 | HeLa | cell lines | T | Cervical cancer |  |
| 31 | HCC-44 | cell lines | T | Lung cancer | Lung |
| 32 | A549 | cell lines | T | Lung cancer |  |
| 33 | Caov3 | cell lines | T | Ovarian cancer | Ovary |
| 34 | OV-90 | cell lines | T | Ovarian cancer |  |
| 35 | OVCAR3 | cell lines | T | Ovarian cancer |  |
| 36 | SK-OV3 | cell lines | T | Ovarian cancer |  |
| 37 | SNU119 | cell lines | T | Ovarian cancer |  |
| 38 | SW626 | cell lines | T | Ovarian cancer |  |
| 39 | AGS | cell lines | T | Gastric cancer | Stomach |
| 40 | Kato III | cell lines | T | Gastric cancer |  |
| 41 | MKN1 | cell lines | T | Gastric cancer |  |
| 42 | MKN74 | cell lines | T | Gastric cancer |  |
| 43 | NCI-N87 | cell lines | T | Gastric cancer |  |
| 44 | SNU5 | cell lines | T | Gastric cancer |  |
| 45 | SNU16 | cell lines | T | Gastric cancer |  |
| 46 | SNU484 | cell lines | T | Gastric cancer |  |
| 47 | SNU601 | cell lines | T | Gastric cancer |  |
| 48 | SNU638 | cell lines | T | Gastric cancer |  |
