# Supplementary material for: Identification of Novel Reference Genes Using Multiplatform Expression Data and Their Validation for Quantitative Gene Expression Analysis
Source: PLoS One. 2009 Jul 7;4(7):e6162. doi: 10.1371/journal.pone.0006162 (PMC2703796; doi:10.1371/journal.pone.0006162)
Supplement: Table S4 — Correlation of gene expression and CV of 2,087 candidate HKGs between the four datasets (0.05 MB DOC) [file pone.0006162.s006.doc]

**Table S4.** Correlation of gene expression and CV of 2,087 candidate HKGs between the four datasets

| **Gene expression** |  |  |  |  |  |  |
| --- | --- | --- | --- | --- | --- | --- |
| Datasets | EST-ShortSAGE | EST-LongSAGE | EST-Affy | ShortSAGE-LongSAGE | ShortSAGE -Affy | LongSAGE- Affy |
| Pearson correlation | 0.54 | 0.581 | 0.476 | 0.909 | 0.56 | 0.615 |
| *P* value | <0.001 | <0.001 | <0.001 | <0.001 | <0.001 | <0.001 |
| Spearman correlation | 0.594 | 0.613 | 0.339 | 0.853 | 0.397 | 0.402 |
| *P* value | <0.001 | <0.001 | <0.001 | <0.001 | <0.001 | <0.001 |
| **CV (%)** |  |  |  |  |  |  |
| Pearson correlation | 0.22 | 0.07 | 0.107 | 0.513 | 0.301 | 0.309 |
| *P* value | <0.001 | 0.001 | <0.001 | <0.001 | <0.001 | <0.001 |
| Spearman correlation | 0.223 | 0.074 | 0.19 | 0.456 | 0.389 | 0.324 |
| *P* value | <0.001 | 0.001 | <0.001 | <0.001 | <0.001 | <0.001 |
