# Supplementary material for: Identification of Novel Reference Genes Using Multiplatform Expression Data and Their Validation for Quantitative Gene Expression Analysis
Source: PLoS One. 2009 Jul 7;4(7):e6162. doi: 10.1371/journal.pone.0006162 (PMC2703796; doi:10.1371/journal.pone.0006162)
Supplement: Table S5 — CpG islands analysis in the upstream region from transcription start site of 13 nERGs (0.04 MB DOC) [file pone.0006162.s007.doc]

**Table S5.** CpG islands analysis in the upstream region from transcription start site of 13 nERGs

| **Gene Symbol** |  | **Refseq** | **CpG island*** | |  |  |  |
| --- | --- | --- | --- | --- | --- | --- | --- |
|  |  |  | **Start** | **End** | **GC%** | **O/E(CpG)** | **Length(bp)** |
| OAZ1 |  | NM_004152.2 | -650 | -2 | 59.4 | 1 | 649 |
| UBQLN1 |  | NM_013438.3 NM_053067.1 | -551 | -2 | 56.6 | 0.73 | 550 |
| GPBP1 |  | NM_022913.1 | -1003 | -2 | 63.2 | 0.9 | 1002 |
| CTBP1 |  | NM_001328.2 NM_001012614.1 | -1211 | -2 | 70.6 | 0.86 | 1210 |
| PAPOLA |  | NM_032632.3 | -850 | -2 | 60.8 | 0.92 | 849 |
| ARL8B |  | NM_018184.2 | -343 | 962 | 57.7 | 0.92 | 1305 |
| SPG21 |  | NM_016630.3 | -506 | -2 | 55.1 | 0.81 | 505 |
| LUC7L2 |  | NM_016019.2 | -1191 | -2 | 60.1 | 0.91 | 1190 |
| ZNF207 |  | NM_001098507.1 NM_001032293.2 NM_003457.3 | -1240 | -537 | 55 | 0.68 | 704 |
| DIMT1L |  | NM_014473.2 | -781 | -253 | 55.1 | 0.7 | 529 |
| TRIM27 |  | NM_006510.4 | -507 | -3 | 61.5 | 0.65 | 505 |
| CUL1 |  | NM_003592.2 | -917 | -2 | 65.5 | 0.89 | 916 |
| FBXW2 |  | NM_012164.3 | -480 | 660 | 57 | 0.84 | 1140 |

Upstream sequences (2000 bp from transcription start site) were downloaded from the UCSC site

(http://hgdownload.cse.ucsc.edu/goldenPath/hg18/bigZips/).

*****CpG island criteria: length ≥ 500 bp, % GC ≥ 55, CpG o/e ratio≥0.65
