# Supplementary material for: Identification of Novel Reference Genes Using Multiplatform Expression Data and Their Validation for Quantitative Gene Expression Analysis
Source: PLoS One. 2009 Jul 7;4(7):e6162. doi: 10.1371/journal.pone.0006162 (PMC2703796; doi:10.1371/journal.pone.0006162)
Supplement: Table S6 — Correlation of gene expression and CV of 13 nERGs between the four datasets (0.05 MB DOC) [file pone.0006162.s008.doc]

**Table S6.** Correlation of gene expression and CV of 13 nERGs between the four datasets

| **Gene expression** |  |  |  |  |  |  |
| --- | --- | --- | --- | --- | --- | --- |
| Datasets | EST-ShortSAGE | EST-LongSAGE | EST-Affy | ShortSAGE-LongSAGE | ShortSAGE -Affy | LongSAGE- Affy |
| Pearson correlation | 0.951 | 0.978 | 0.834 | 0.974 | 0.843 | 0.869 |
| *P* value | <0.001 | <0.001 | <0.001 | <0.001 | <0.001 | <0.001 |
| Spearman correlation | 0.731 | 0.698 | 0.374 | 0.714 | 0.511 | 0.56 |
| *P* value | 0.006 | 0.010 | 0.206 | 0.008 | 0.076 | 0.049 |
| **CV (%)** |  |  |  |  |  |  |
| Pearson correlation | -0.319 | -0.463 | 0.145 | 0.338 | 0.209 | 0.082 |
| *P* value | 0.288 | 0.111 | 0.636 | 0.259 | 0.494 | 0.791 |
| Spearman correlation | -0.396 | -0.5 | 0.184 | 0.258 | -0.025 | 0.033 |
| *P* value | 0.182 | 0.085 | 0.547 | 0.389 | 0.936 | 0.915 |
