# Supplementary material for: Identification of Novel Reference Genes Using Multiplatform Expression Data and Their Validation for Quantitative Gene Expression Analysis
Source: PLoS One. 2009 Jul 7;4(7):e6162. doi: 10.1371/journal.pone.0006162 (PMC2703796; doi:10.1371/journal.pone.0006162)
Supplement: Table S7 — tERGs used in this study (0.05 MB DOC) [file pone.0006162.s009.doc]

**Table S7.** tERGs used in this study

| **UniGene cluster** | **Gene Symbol** | **Gene Title** | **EST** | | | **SHORT SAGE** | | | **LONG SAGE** | | | **Affymetrix** | |
| --- | --- | --- | --- | --- | --- | --- | --- | --- | --- | --- | --- | --- | --- |
|  |  |  | Mean | CV | 0's P | Mean | CV | 0's P | Mean | CV | 0's P | Mean | CV |
| Hs.448226 | RPLP0 | Ribosomal protein, large, P0 | 3809.2 | 75.92 | 0 | 1108.65 | 91.01 | 0 | 1605.76 | 74.12 | 0 | 1888.46 | 41.47 |
| Hs.520640 | ACTB | β-actin | 4381.34 | 95.98 | 0.034 | 1348.49 | 91.02 | 0 | 1961.29 | 60.59 | 0.111 | 5036.63 | 31.42 |
| Hs.356331 | PPIA | Peptidylprolyl isomerase A (cyclophilin A) | 1225.7 | 78.02 | 0.034 | 1646.83 | 59.53 | 0 | 1683.27 | 56.79 | 0 | 5223.43 | 28.46 |
| Hs.479728 | GAPDH | Glyceraldehyde-3-phosphate dehydrogenase | 7330.72 | 80.18 | 0 | 3167.05 | 83.62 | 0 | 3178.15 | 102.5 | 0 | 4934.56 | 42.86 |
| Hs.78771 | PGK1 | Phosphoglycerate kinase 1 | 681.19 | 86.72 | 0.034 | 423.67 | 85.52 | 0 | 445.96 | 90.9 | 0 | 1179.63 | 41.05 |
| Hs.534255 | B2M | β-2-microglobulin | 1303.98 | 172.16 | 0 | 2594.12 | 96.87 | 0 | 2910.69 | 103.61 | 0 | 4736.9 | 32.54 |
| Hs.255230 | GUSB | β-Glucuronidase | 116.77 | 89.37 | 0.414 | 40.98 | 67.81 | 0.107 | 11.42 | 89.36 | 0.556 | 360.37 | 49.14 |
| Hs.412707 | HPRT1 | Hypoxanthine phosphoribosyltransferase 1 | 103.48 | 63.18 | 0.345 | 32.51 | 63.51 | 0.107 | 33.29 | 49.07 | 0.222 | 233.36 | 40.85 |
| Hs.1100 | TBP | TATA box binding protein | 71.46 | 47.77 | 0.448 | 31.94 | 62.8 | 0.286 | 17.58 | 69.22 | 0.444 | 25.44 | 73.23 |
| Hs.529618 | TFRC | Transferrin receptor (p90, CD71) | 212.76 | 85.52 | 0.241 | 89.51 | 87.09 | 0.036 | 82.62 | 91.59 | 0.111 | 36.01 | 56.84 |
| Hs.82609 | HMBS | Hydroxymethylbilane synthase | 176.26 | 105.68 | 0.172 | 32.51 | 76.51 | 0.214 | 28.04 | 42.5 | 0.444 | 119.08 | 33.63 |
| Hs.463511 | H6PD | Hexose-6-phosphate dehydrogenase (glucose-1-dehydrogenase) | 101.9 | 86.9 | 0.483 | 44.65 | 70.8 | 0.071 | 25.33 | 101.45 | 0.222 | 25.04 | 28.85 |
| Hs.476308 | ALAS1 | δ-Aminolevulinate synthase 1 | 132.82 | 82.5 | 0.345 | 50.43 | 72.23 | 0 | 22.5 | 50.59 | 0.222 | 178.95 | 107.93 |

Mean: Mean gene expression, CV: Coefficient of Variation (%), 0’s P: 0’s proportion
