# Supplementary material for: Identification of Novel Reference Genes Using Multiplatform Expression Data and Their Validation for Quantitative Gene Expression Analysis
Source: PLoS One. 2009 Jul 7;4(7):e6162. doi: 10.1371/journal.pone.0006162 (PMC2703796; doi:10.1371/journal.pone.0006162)
Supplement: Table S8 — Comparison of CV between nERGs and tERGs in the dataset (0.04 MB DOC) [file pone.0006162.s010.doc]

**Table S8.** Comparison of CV between nERGs and tERGs in the dataset

|  | **nERGs (n=13)*** | **tERGs (n=13)*** | ***P* value **** |
| --- | --- | --- | --- |
| EST | 61.12± 5.93 | 88.45± 28.92 | <0.001 |
| ShortSAGE | 60.64±3.10 | 77.56±12.37 | <0.001 |
| LongSAGE | 49.82±4.12 | 75.56±22.18 | 0.003 |
| Affy | 25.85±3.09 | 46.79±22.22 | <0.001 |
| *Mean±standard deviation | | | |
| ** Wilcoxon rank sum test | | | |
