# Supplementary material for: Identification of Novel Reference Genes Using Multiplatform Expression Data and Their Validation for Quantitative Gene Expression Analysis
Source: PLoS One. 2009 Jul 7;4(7):e6162. doi: 10.1371/journal.pone.0006162 (PMC2703796; doi:10.1371/journal.pone.0006162)
Supplement: Table S9 — Comparison of Cp values between nERGs and tERGs in qRT-PCR (0.04 MB DOC) [file pone.0006162.s011.doc]

**Table S9.** Comparison of Cp values between nERGs and tERGs in qRT-PCR

|  |  | **tERGs*** | **nERGs (n=13)*** | ***P* value**** |
| --- | --- | --- | --- | --- |
| **48 frozen tissues/cell lines** | High tERGs(n=4)^a^ | 17.84±1.82 | 22.84±1.55 | <0.001 |
|  | Low tERGs(n=3)^b^ | 24.54±1.73 | 22.84±1.55 | <0.001 |
| **60 FFPE tissues** | High tERGs(n=3)^c^ | 22.76±1.49 | 26.84±1.47 | <0.001 |
|  | Low tERGs(n=4)^d^ | 29.40±1.26 | 26.84±1.47 | <0.001 |
| *Mean ± standard deviation | | | | |
| ** Wilcoxon rank sum test  ^a^High tERGs include *GAPDH, ACTB, B2M, PPIA*, ^b^Low tERGs include *HPRT1, HMBS, TBP.*  ^c^High tERGs include *GAPDH, ACTB, B2M*, ^d^Low tERGs include *PPIA,HPRT1, HMBS,TBP.* | | | | |
