# Supplementary material for: Identification of Novel Reference Genes Using Multiplatform Expression Data and Their Validation for Quantitative Gene Expression Analysis
Source: PLoS One. 2009 Jul 7;4(7):e6162. doi: 10.1371/journal.pone.0006162 (PMC2703796; doi:10.1371/journal.pone.0006162)
Supplement: Table S10 — Comparison of gene expression stability values between nERGs and tERGs (0.04 MB DOC) [file pone.0006162.s012.doc]

**Table S10.** Comparison of gene expression stability values between nERGs and tERGs

|  |  | **nERGs (n=13)*** | **tERGs (n=7)*** | ***P* value **** |
| --- | --- | --- | --- | --- |
| **48 frozen tissues/cell lines** | M | 0.616±0.083 | 0.760±0.117 | 0.014 |
|  | S | 0.372±0.085 | 0.541±0.174 | 0.032 |
|  |  | **nERGs (n=12)***** | **tERGs (n=7)*** | ***P* value **** |
| **60 FFPE tissues** | M | 0.509±0.072 | 0.663±0.057 | 0.005 |
|  | S | 0.299±0.064 | 0.462±0.071 | 0.027 |
| *Mean±standard deviation | | | | |
| ** Wilcoxon rank sum test | | | | |
| ***DIMT1L was excluded in the analysis | | | | |
| M: average expression stability calculated by the geNorm program, S: stability value calculated by the NormFinder progrm | | | | |
